# Donnan‐Engineered Inner Helmholtz Plane Enabling Ultra‐Stable Aqueous Bismuth Electrode

**DOI:** 10.1002/advs.202508965

**Published:** 2025-07-29

**Authors:** Tingting Qin, Wenli Zhang, Dong Wang, Yingguang Zhang, Taowen Dong, Zibo Zhang, Sarah K.W. Leong, Weichen Chen, Samy Mahmoud, Hanqing Liu, Yifei Wang, Xiaolong Zhao, Wei Dong, Meng Ni, Dennis Y.C. Leung, Zhenbin Guo, Wending Pan

**Affiliations:** ^1^ Department of Mechanical Engineering The University of Hong Kong Hong Kong 999077 China; ^2^ Institute of Semiconductor Manufacturing Research Shenzhen University Shenzhen 518060 China; ^3^ School of Chemical Engineering and Light Industry Guangdong University of Technology Guangzhou 510006 China; ^4^ Department of Building and Real Estate The Hong Kong Polytechnic University Hong Kong 999077 China; ^5^ Key Laboratory of Automobile Materials of MOE School of Materials Science and Engineering and Jilin Provincial International Cooperation Key Laboratory of High‐Efficiency Clean Energy Materials Jilin University Changchun 130013 China; ^6^ Department for Electrochemical Energy Storage Helmholtz‐Zentrum Berlin für Materialien und Energie Hahn‐Meitner Platz 114109 Berlin Germany

**Keywords:** aqueous metal electrode, bismuth, donnan effect, inner helmholtz plane, ion‐sieving and reaction‐sieving

## Abstract

The electrochemical instability of the solid‐liquid interface remains a critical bottleneck in rechargeable aqueous metal batteries (RAMBs), where traditional strategies fail to resolve the inherent conflict between electrochemical and parasitic reactions at the inner Helmholtz plane (IHP). Herein, inspiring from the ion‐sieving principles of the Donnan effect, classical electrostatics is integrated with interfacial engineering by creating a phosphate anion (PO_4_^3−^)‐adsorbed IHP on a bismuth (Bi) electrode. The immobilized PO_4_^3−^ establishes a sustained Donnan potential, driving three key functions: i) ion and reaction sieving through charge‐selective ion partitioning, enriching Na⁺ while excluding OH⁻ to enable selective (de)alloying over corrosion reaction; ii) electron confinement through Donnan potential to suppress parasitic electron leakage; and iii) dynamic stabilization of the IHP through strong anion chemisorption, bridging the classical Donnan model with electrochemistry. As a result, the Bi electrode demonstrates a superior cycling stability (200 mAh g^−1^ retention after 3000 cycles at 2 A g^−1^) and ultrahigh‐rate performance (134 mAh g^−1^ at 16 A g^−1^). By extending the Donnan effect in electrochemistry, the research creates a universal interfacial paradigm based on charge‐selective ion partitioning and electron confinement. This breakthrough provides a transformative strategy to decouple desired electrochemical reactions from parasitic side reactions, paving the way for advanced RAMBs.

## Graphical abstract

A Donnan‐effect‐mimetic inner Helmholtz plane (IHP) can stabilize aqueous Bismuth electrodes through two synergistic mechanisms: i) charge‐selective ion partitioning that enriches Na⁺ while excluding OH⁻, enabling preferential (de)alloying over corrosive side reactions; and ii) electron confinement via the Donnan potential, which effectively suppresses parasitic charge leakage.

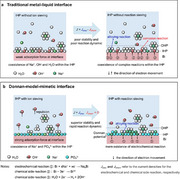

## 1 Introduction

Rechargeable aqueous metal batteries (RAMBs) have gained significant attention in both academia and industry owing to their high theoretical energy density and inherent non‐flammability.^[^ ^1^ ^]^ However, their practical application is hampered by inadequate cycling stability, primarily attributed to the chemical and structural instability at the interface between the metal anode (e.g., Pb, Zn, Mg, Bi) and electrolyte during charging and discharging processes.^[^ ^1^, ^2^ ^]^ In particular, the direct exposure of the highly reactive metals to the aqueous electrolytes leads to the formation of a highly unstable interfacial environment. This instability triggers two major issues: i) parasitic chemical side reactions (including the hydrogen evolution reaction (HER), corrosion, and passivation); ii) irreversible electrochemical reactions, such as zinc metal dendrite growth.^[^ ^3^ ^]^

To address this challenge, research efforts have primarily focused on stabilizing the solid‐liquid interfaces through structural optimization of electrodes and chemical regulation of electrolyte systems. In metal electrode design, structural engineering approaches aim to promote uniform nucleation kinetics and optimize subsequent deposition processes. This encompasses 3D electrode architectures^[^ ^4^ ^]^ and artificial solid electrolyte interphases (ASEI),^[^ ^5^ ^]^ as well as chemical modifications like varying alloy compositions.^[^ ^6^ ^]^ Parallel efforts in electrolyte innovation employ diverse formulations^[^ ^7^ ^]^ ranging from high‐concentration^[^ ^8^ ^]^ to low‐concentration^[^ ^9^ ^]^ and gel‐based systems,^[^ ^10^ ^]^ –along with functional additives to modulate the solvation environment of metal ions^[^ ^11^ ^]^ and minimize free water activity.^[^ ^12^ ^]^ These synergistic modifications collectively enable stabilized metal deposition processes, effective suppression of parasitic reactions, and expansion of electrochemical operational windows.^[^ ^13^ ^]^ Despite these advancements, RAMBs have not yet met industry needs, especially for long‐term and high‐rate operations.^[^ ^3b^ ^]^ The primary limitation stems from their inability to resolve the fundamental selectivity conflict between chemical and electrochemical reactions at the Inner Helmholtz Plane (IHP) during charging and discharging cycles–this critical interface governs both ion transport and charge transfer processes.^[^ ^14^ ^]^

As the core region for charge transfer and ion exchange, the ion arrangement within the IHP is governed by the potential‐dependent adsorption affinity of interface species and the electric field from electrode polarization.^[^ ^15^ ^]^ The traditional chemical composition of the electric double layer (EDL) for metal electrodes follows the *Gouy‐Chapman‐Stern* (GCS) model. The chemical composition within the IHP is inherently complex, with randomly distributed ions that may not actively participate in the desired electrochemical storage reactions,^[^ ^16^ ^]^ their presence introduces significant challenges. For instance, water molecules and certain interfering ions can often trigger unwanted chemical side reactions.^[^ ^15^ ^]^ This complex arrangement within the IHP leads to intricate interfacial reactions that couple chemical and electrochemical processes. Moreover, the dynamic charging and discharging processes induce continuous restructuring of IHP chemistry, driven by dynamic changes in the metal surface state (such as oxidation/reduction, phase transitions). Such fluctuations disrupt the metastable equilibrium between key components within the IHP–the metal electrode substrate, target storage ions, and interfering ions–initiating chemical parasitic reactions and self‐discharge, ultimately leading to reduced cycle stability.

The Donnan effect is a classical theory describing imbalanced ion distribution induced by fixed charges. This theory provides crucial insights for interface engineering design: when immobile fixed charges are present in the interfacial region, the enrichment of counterions and exclusion of co‐ions will jointly act through establishing a local electrical potential gradient to form selective ion transport pathways.^[^ ^17^ ^]^ However, the traditional Donnan model primarily addresses static membrane separation systems (such as ion exchange membranes),^[^ ^18^ ^]^ and its application to dynamic electrochemical IHP, has been largely overlooked. Establishing a mechanism to regulate the permeability and selectivity of specific ions within the dynamic IHP during electrochemical reaction is essential for ensuring a stable interfacial reaction. By strategically incorporating the Donnan effect to modulate the surface charge and potential distribution of the IHP, ion selectivity (either by excluding or allowing specific ions) and ion/electron transport dynamics can be optimized. This strategy could serve as an effective solution for stabilizing the metal‐electrolyte interface for RAMBs.

In this study, we introduce a novel extension of the Donnan model to resolve the fundamental selectivity conflict between chemical and electrochemical reactions within IHP. The Bi metal system was chosen as a proof‐of‐concept primarily due to the close proximity of the potentials between the electrochemical alloying reaction and the parasitic chemical corrosion reaction. This allows for an ideal validation of the Donnan model's role in selecting between electrochemical and chemical processes in IHP. A Donnan‐mimetic IHP, featuring a strongly adsorbed PO_4_^3−^ layer, was designed to serve as the fixed‐charge functionality of classical Donnan membranes. This rationally designed IHP architecture achieves the following key breakthroughs: i) ion‐sieving and reaction‐sieving functionality: the fixed negative charges of the PO_4_^3−^ layer induce a Donnan potential, driving Na⁺ (storage counter‐ions) to accumulate in the IHP for desired for alloying and dealloying reaction, while repelling OH⁻ (interfering co‐ions), suppressing undesired corrosion reactions; and ii) electron confinement functionality: the PO_4_^3−^‐adsorbed IHP generates a Donnan potential that suppresses parasitic electron leakage from Bi to the electrolyte, further preventing self‐discharge. This study establishes a groundbreaking conceptual framework that extends the theory of the Donnan model to dynamic electrochemical systems. By resolving the dynamic coordination between electron confinement and ion partitioning at the molecular‐level IHP, it lays the foundation for universal strategies to construct highly stable solid‐liquid interfaces for RAMBs.

## 2 Results and Discussion

### 2.1 Design of Donnan‐Effect‐Mimetic IHP

**Figure 1:**
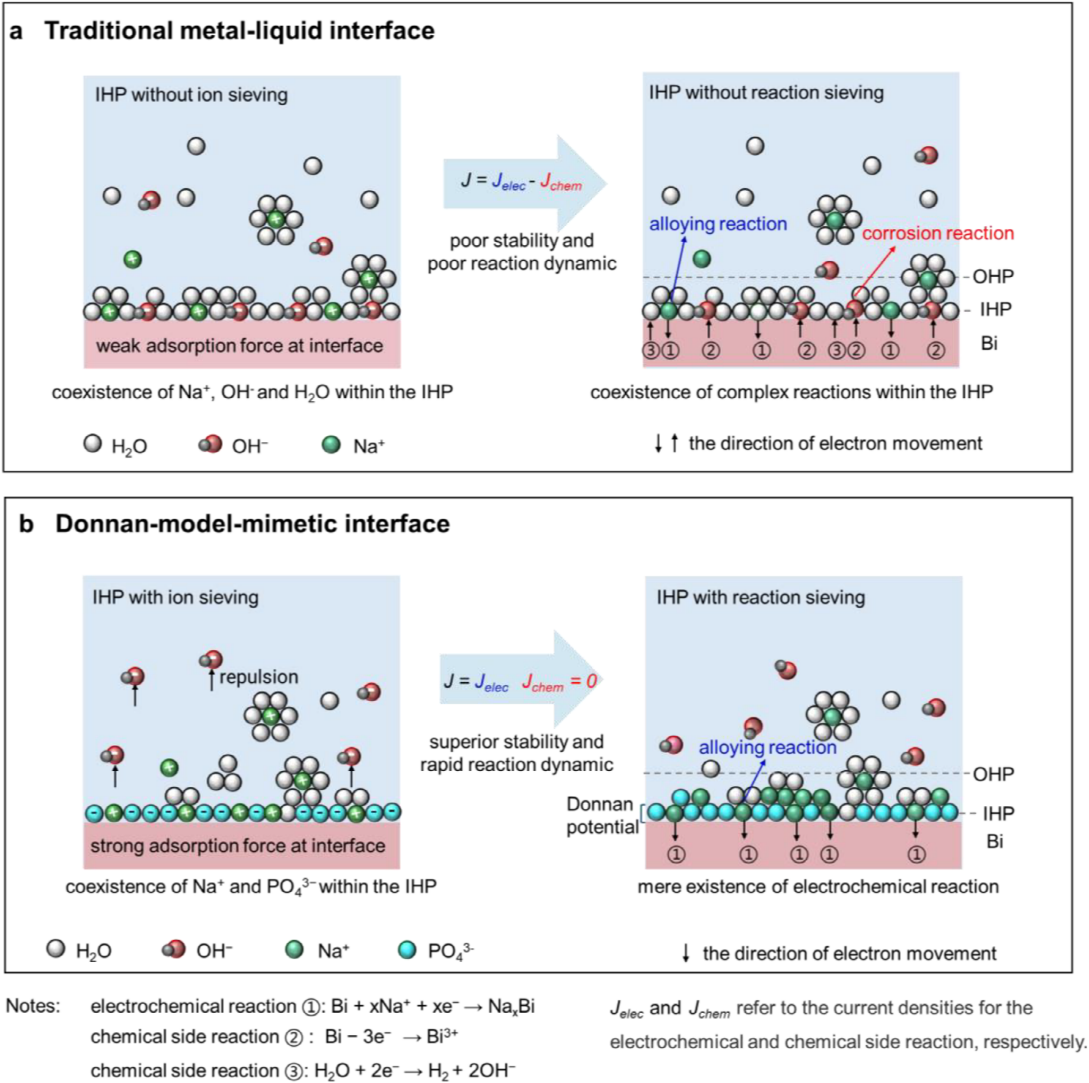
Design concept of Donnan‐model‐mimetic IHP. a) Traditional solid‐liquid interface lacking Donnan charge screening, exhibiting chaotic IHP compositions (H_2_O/Na^+^/OH^−^) and coupled electrochemical and chemical reactions; b) Donnan‐model‐mimetic IHP through molecularly adsorbed PO_4_^3−^ anions, which establish ion‐sieving and reaction‐sieving functionalities. Notes: The electrochemical reaction is given by ①: Bi + xNa^+^ +xe^−^ ↔ Na_x_Bi, while the chemical side reactions are ②: Bi − 3e^−^ → Bi^3+^ (corrosion reaction) and ③: 2H_2_O + 2e^−^ → H_2_ + 2OH^−^ (HER reaction). IHP and OHP denote the interfacial regions of the Inner and Outer Helmholtz Planes, respectively, while *J_elec_* and *J_chem_* represent the current densities associated with the electrochemical and chemical reaction pathways.

Conventional Bi electrodes exhibit chaotic IHP compositions (H_2_O/Na^+^/OH^−^) that permit simultaneous occurrence of: ① Electrochemical (de)alloying: ①: Bi + xNa^+^ +xe^−^ ↔ Na_x_Bi;^[^ ^19^ ^]^ ② Chemical corrosion: Bi − 3e^−^ → Bi^3+^; ③ Residual HER: 2H_2_O + 2e^−^ → H_2_ + 2OH^−^ (**Figure** **1**a).^[^ ^20^ ^]^ The missing Donnan potential allows OH⁻ penetration that not only consumes electrons through Reaction ② (self‐discharge) but also sterically hinders Na⁺‐Bi alloy formation. This results in compromised interfacial current density (*J = J_elec_ – J_chem_*) and rapid capacity decay.

To address this, we engineer a Donnan‐effect‐mimetic IHP to reconstruct the composition of chemical species within IHP, thereby regulating the interfacial reactions (Figure 1b). By leveraging the strong chemical adsorption force between PO_4_^3−^ and Bi metal, PO_4_^3−^ was fixed on the Bi metal surface, occupying the position of the IHP. This negatively charged Donnan‐effect‐mimetic IHP serves two functions: i) it adsorbs positively charged Na^+^ closer to the Bi while excluding OH⁻, enhancing the alloying reaction (*J_elec_* is enhanced); ii) the fixed PO_4_^3−^ generate a Donnan potential, which suppresses the outward electron flow from the metal to the electrolyte (*J_chem_* = 0). This, in turn, inhibits the electron‐loss process of Bi metal (corrosion reaction) and the electron‐gain process of H_2_O (HER reaction). As a result, the overall interfacial exchange current is significantly enhanced compared to the system without the Donnan‐effect‐mimetic IHP, leading to improvements in both stability and rate capability.

Based on the aforementioned hypothesis of creating a Donnan‐model‐mimetic IHP for the Bi electrode, we incorporated Na_3_PO_4_ into the NaOH electrolyte, naming this modified solution BEP. BEP was prepared by mixing NaOH (1 M) with Na_3_PO_4_·12H_2_O solution (0.333 M) in a volume ratio of 25:1. In contrast, the baseline electrolyte without PO_4_^3−^, is referred to as BE (see details in the Experimental Section). The Bi metal was synthesized via a facile reduction reaction on a Ni substrate, forming densely packed large single crystals with dimensions ranging from 3 to 5 µm that are uniformly distributed across the substrate surface. Structural characterization reveals that the synthesized Bi exhibits excellent crystallinity, predominantly exposing the (012) crystallographic plane, and adopts a well‐defined layered hexagonal structure. (see preparation details in the Experimental Section and structural and morphological characterizations in Figures S1–S3, Supporting Information).

### 2.2 Physicochemical Characterization of Donnan‐Effect‐Mimetic IHP

**Figure 2:**
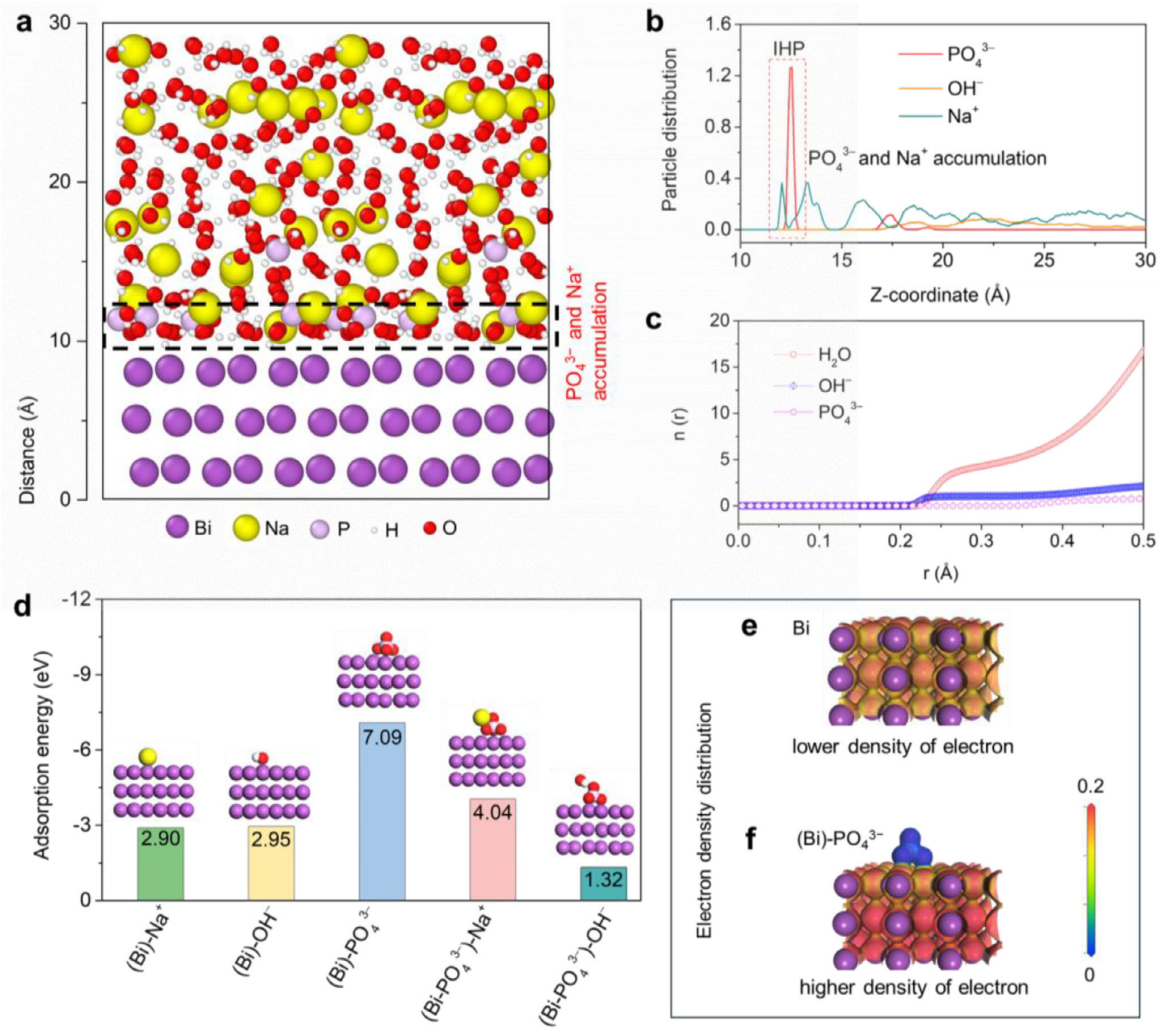
Theoretical characterization for Donnan‐effect‐mimetic IHP. a) Illustration of the distribution for the different chemical species within the EDL; b) Statistical particle distribution at the Bi(012)/electrolyte interface; c) Coordination particles around Na^+^ and related statistical coordination number; d) Adsorption energies of the selected ions adsorbed on the Bi plane and (Bi‐PO_4_^3−^); Electron density distribution for e) Bi and f) (Bi)‐PO_4_^3−^.

The Donnan‐effect‐mimetic IHP can achieve ion‐sieving functionality, as confirmed through both theoretical and experimental characterization. Molecular dynamics (MD) simulations demonstrated how the Donnan effect regulates ion partitioning within the EDL for the Bi‐BEP electrode (with the addition of PO_4_^3^⁻). In the simulated BEP electrolytes, a snapshot (**Figure** **2**a) revealed that each component was uniformly distributed in the simulated box without any phase separation, suggesting that PO_4_^3^⁻ was well dissolved in the electrolyte. Its preferential accumulation in the innermost EDL layer, directly adjacent to the Bi surface, was observed. The large, multivalent PO_4_^3^⁻ anions formed a densely packed screening layer at the IHP, creating a charge‐selective barrier that mimics the semi‐permeable membrane behavior seen in classical Donnan equilibria.^[^ ^21^ ^]^

The distinct spatial stratification within EDL (Figure 2b; Figure S4, Supporting Information) further evidenced this Donnan‐type ion‐sieving mechanism: PO_4_^3^⁻ dominated the primary adsorption layer (offset from smaller Na⁺ ions due to steric and charge‐density effects), followed by a secondary layer of Na⁺ and H_2_O. OH⁻ were expelled to outer regions due to Donnan repulsion from the concentrated PO_4_^3−^ layer, effectively suppressing their access to the electrode surface and thereby mitigating parasitic corrosion reactions. Coordination analysis (Figure 2c) confirms Na⁺ solvation with ≈4.5 H_2_O molecules and PO_4_^3^⁻ association (CN ≈ 0.3), aligning with Donnan‐driven ion partitioning. The high electrostatic potential near the IHP preferentially stabilizes multivalent PO_4_^3^⁻ over monovalent OH⁻/Na⁺, while hydrated Na⁺ remains mobile in the diffuse layer. This Donnan‐mimetic architecture establishes a charge‐selective interface: PO_4_^3^⁻ acts as a steric‐electrostatic filter, enabling selective Na⁺ transport while excluding OH⁻–a pivotal feature for stabilizing Bi electrodes against unwanted chemical corrosion.

Experimental validation via Time‐of‐Flight secondary ion mass spectrometry (TOF‐SIMS) in Figures S5 and S6 (Supporting Information) and P2p X‐ray photoelectron spectroscopy (XPS) in Figure S7 (Supporting Information), reveals uniform P distribution on the Bi surface. In the Bi‐BEP electrode, the Bi^0^ 4f peaks (4f_7/2_ at 156.4 eV and 4f_5/2_ at 161.7 eV) coexist with Bi^3+^ peaks (4f_7/2_ at 158.16 eV and 4f_5/2_ at 163.46 eV), attributed to electron sharing between Bi and PO_4_^3−^,^[^ ^22^ ^]^ inducing a positive shift relative to metallic Bi^0^. This electronic interaction confirms PO_4_^3−^ chemisorption,^[^ ^23^ ^]^ reinforcing the Donnan‐stabilized interfacial architecture (Figures S8 and S9, Supporting Information).

The stratified ion partitioning in the EDL results from the Donnan equilibrium established between the fixed PO_4_^3^⁻ anions within the IHP and the mobile ions (Na⁺/OH⁻) in the OHP/diffuse layer. To quantify these interfacial interactions, the adsorption energies were calculated using Equation 1:

(1) $$\Delta E_{ads}=E_{sur+ion}\;-\;E_{sur}\;-\;E_{ion}$$

where *E_sur_* denotes the energy of either the pristine Bi(012) surface or the PO_4_^3^⁻‐adsorbed Bi(012) surface (denoted as Bi‐PO_4_^3^⁻); *E*_*sur*  +  *ion*_ represents the total energy of Bi or Bi‐PO_4_^3^⁻ after the subsequent adsorption of ions; *E_ion_* is the energy of the selected ions. The optimized adsorption configurations and their respective energy values were presented in Figure 2d.

The more negative of the adsorption energy, the stronger the adsorption ability. Based on the calculated adsorption energies, it is evident that Bi metal exhibits a significantly stronger affinity toward PO_4_^3^⁻ (−7.09 eV) compared to Na^+^ (−2.95 eV) and OH^−^ (−2.90 eV). This observation suggests that Bi metal preferentially adsorbs PO_4_^3^⁻, aligning with aforementioned MD findings and XPS results that indicate the predominant of PO_4_^3^⁻ within the IHP. The close values of Bi metal toward Na^+^ and OH^−^ also elucidate the occurrence of the inevitable corrosion reaction during the alloying process. Crucially, constructing a PO_4_^3^⁻‐adsorbed Bi surface (Bi‐PO_4_^3^⁻) induces a Donnan potential barrier that fundamentally reverses ion selectivity: Na⁺ adsorption strengthens (−4.04 eV) while OH⁻ interaction weakens (−1.32 eV). Electron density analysis confirms this selectivity mechanism, revealing Bi‐O covalent bonding between the O of PO_4_^3^⁻ and Bi (Figure 2e,f), which enhances interfacial negative charge density compared to bare Bi.

This charge redistribution amplifies the Donnan potential, establishing a charge‐asymmetric EDL. Within this architecture, immobilized PO_4_^3^⁻ anions act as molecular‐scale Donnan filters–electrostatically concentrating Na⁺ counterions at the IHP while repelling OH⁻ co‐ions to the OHP and diffuse layer. The resultant ion‐sieving effect not only facilitates Na⁺ migration but also effectively blocks OH⁻ penetration during electrochemical cycling, thereby substantially reducing corrosive side reactions and establishing a reaction‐sieving mechanism.

### 2.3 Electrochemical Effects of the Donnan‐Effect‐Mimetic IHP

**Figure 3:**
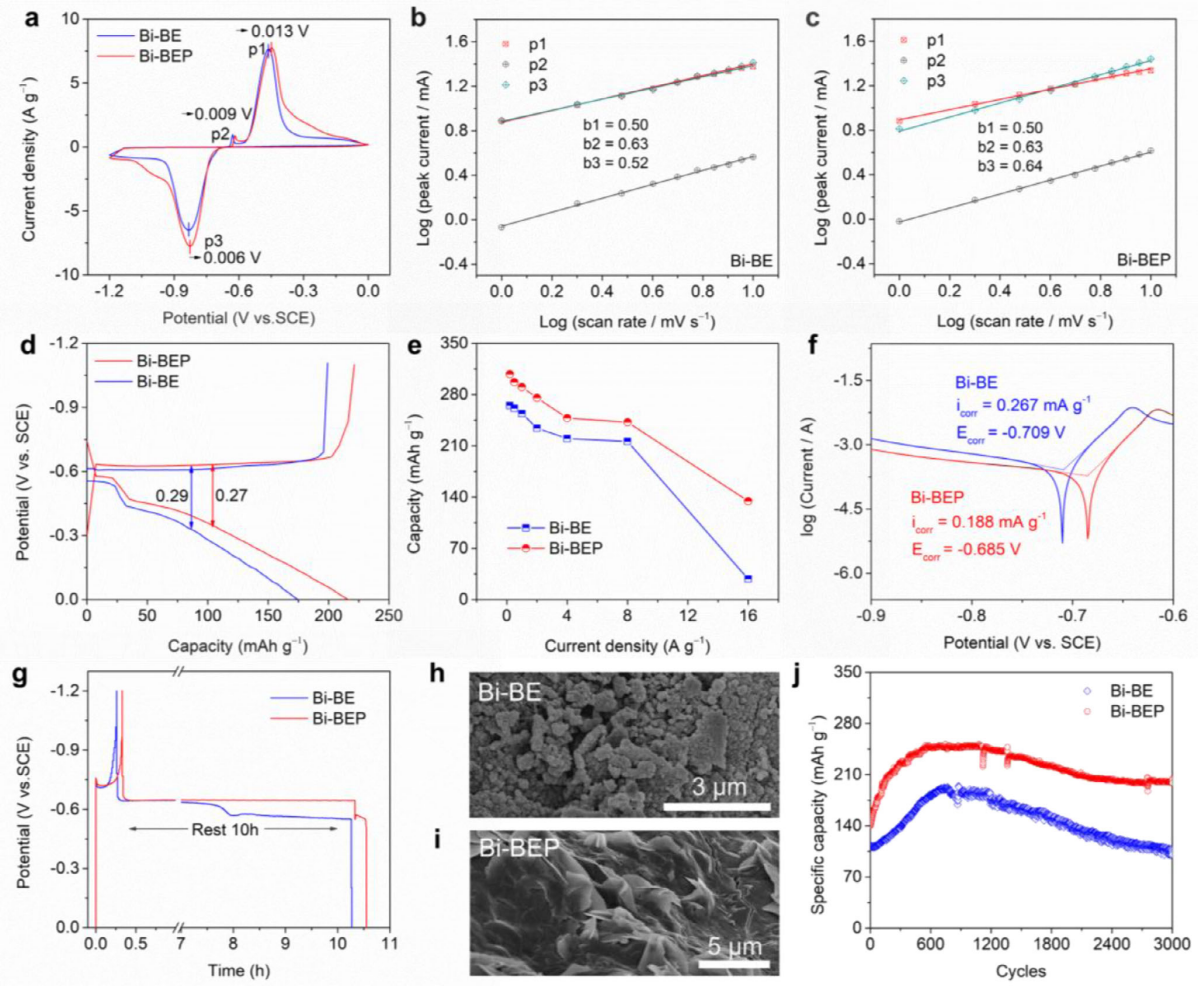
Electrochemical effects of the Donnan‐effect‐mimetic IHP. a) CV curves at a scan rate of 1 mV s^−1^; b‐c) Plots of *log(i)* versus *log(v)* from CV curves at different scan rates; d) GCD curves for Bi‐BE and Bi‐BEP electrodes at a current density of 2 A g^−1^; e) Rate performance at different densities; f) Linear polarization curves of anodic corrosion in the BE and BEP; g) Self‐discharge curves at a current density of 1 A g^−1^; SEM image for h) Bi‐BE and i) Bi‐BEP electrode after 200 cycles; j) Cycling stability at a current density of 2 A g^−1^ for 3000 cycles.

The PO_4_^3−^‐adsorbed IHP for Bi‐BEP profoundly influences the Faradaic processes, by leveraging Donnan equilibrium principles to modulate interfacial ion partitioning and reaction pathways, and dynamics. As shown in **Figure** **3**a, cyclic voltammetry (CV) curves of Bi‐BEP and Bi‐BE exhibit nearly identical shapes, confirming that PO_4_^3−^ introduction preserves the intrinsic (de)alloying reaction pathways. The Na^+^ storage mechanism of the Bi electrode involves a one‐step alloying (reduction) reaction, where Bi directly forms the thermodynamically stable phase Na₃Bi (P3), with relatively low kinetic barriers. Whereas, the dealloying (oxidation) process occurs in two steps:$\;Na_{3}Bi\overset{P2}{\to }NaBi\overset{P1}{\to }Bi$.^[^ ^24^ ^]^ When Na^+^ is extracted from the Bi lattice matrix, it requires overcoming a higher energy barrier related to interfacial reconstruction and atom diffusion. During the dealloying reaction, a thermodynamically stable NaBi intermediate phase may form and exist transiently (P2).^[^ ^25^ ^]^

The subtle shifts in the overpotentials for these reactions highlight the critical role of the Donnan effect. For the alloying reaction (P3: Bi + 3Na⁺ + 3e⁻ → Na₃Bi), Bi‐BEP shows a 6 mV positive shift compared to Bi‐BE, indicating reduced reaction barriers. This improvement originates from the electronegative PO_4_^3−^‐adsorbed IHP, which establishes a Donnan potential gradient. This gradient selectively concentrates Na⁺ and excludes OH⁻ within the IHP via Coulombic attraction, further lowering the alloying barrier. Conversely, dealloying reactions exhibit right‐shifts of 9 mV (P2: Na₃Bi − 2e⁻ → NaBi + 2Na⁺) and 13 mV (P1: NaBi − e⁻ → Bi + Na⁺), which is attributed to the Donnan potential hindering Na⁺ release by increasing the energy barrier for desorption. In any case, thanks to the PO_4_^3−^‐adsorbed IHP, the Bi‐BEP electrode exhibits significantly higher peak current densities during both alloying and dealloying reactions compared to Bi‐BE. This indicates that the Donnan‐effect‐mimetic IHP can enhance the overall electrochemical reaction dynamics.

To further elucidate charge storage dynamics, we performed a quantitative analysis of the current‐scan‐dependent voltametric responses (Figure S10, Supporting Information) using Equation 2:^[^ ^26^ ^]^

(2) log(i)=blog(v)+log(a)

where the b value distinguishes electron‐transport‐limited pseudocapacitance (*b* → 1) from ion‐diffusion‐controlled bulk processes (*b* → 0.5).^[^ ^27^ ^]^ Both Bi‐BE and Bi‐BEP exhibited characteristic diffusion control (Figure 3b,c), confirming that solid‐state phase transformations govern the (de)alloying peaks (with *b* ≈ 0.5 for peaks P1‐P3). Crucially, the Donnan‐effect‐mimetic IHP preserves bulk reactions while synergistically enhancing interfacial Na⁺ flux, as evidenced by invariant *b* values. This mechanistic decoupling demonstrates interfacial transport optimization without perturbing the intrinsic alloying and dealloying reaction mechanisms.

Figure 3d presents the Galvanostatic charge‐discharge (GCD) profiles for Bi‐BE and Bi‐BEP at 2 A g^−1^. Both electrodes exhibit battery‐type charging behavior, marked by distinct voltage plateaus, and capacitive‐like discharging characteristics, evidenced by sloped profiles. Notably, Bi‐BEP achieves a higher discharge capacity of 216.1 mAh g^−1^ to Bi‐BE (172.0 mAh g^−1^), alongside a lower polarization potential (0.27 V versus 0.29 V for Bi‐BE). Rate capability was further evaluated across current densities ranging from 0.2 to 16 A g^−1^ (Figure 3e). The Bi‐BEP electrode achieves discharging capacities of 307.8, 296.8, 290.3, 275.6, 247.8, and 242.2 mAh g^−1^ at 0.2, 0.5, 1, 2, 4, and 8 A g^−1^, respectively, significantly outperforming Bi‐BE (265.0, 261.0, 254.1, 234.0, 220.0, and 216.0 mAh g^−1^). The disparity amplifies at higher rates, with Bi‐BEP retaining 134.4 mAh g^−1^ at 16 A g^−1^–4.8 fold higher than Bi‐BE (28.0 mAh g^−1^).

The superiority of Bi‐BEP arises from the Donnan‐effect‐mimetic IHP, which concentrates Na⁺ while electrostatically repelling OH⁻. This microenvironment lowers reaction barriers, maintaining a Na⁺‐rich interface that accelerates alloying dynamics and minimizes polarization. In contrast, Bi‐BE experiences OH⁻/Na⁺ co‐adsorption within the IHP, disrupting the Donnan equilibrium and hindering Na⁺ accessibility, thereby deteriorating reaction dynamics and reducing capacity output.

Linear polarization analysis (Figure 3f) shows that the Bi‐BEP electrode displays a positive shift in corrosion potential (−0.685 V versus −0.709 V for Bi‐BE) and a lower corrosion current density (0.188 mA·g^−1^ versus 0.267 mA·g^−1^ for Bi‐BE). These results indicate that the interfacial stability of the Bi‐BEP electrode has been significantly enhanced in corrosive environments due to the Donnan‐effect‐mimetic IHP. The electronegative PO_4_^3−^‐adsorbed IHP establishes a localized electrostatic barrier that suppresses corrosive ion (e.g., OH⁻) penetration, thereby stabilizing the Bi metal–electrolyte interface and mitigating parasitic corrosion. Self‐discharge behavior was further probed by charging both electrodes to −1.2 V at 1 A g^−1^, followed by a 10‐h resting period and subsequent discharge to 0 V (Figure 3g). The Bi‐BEP electrode retains 67.7% of its initial capacity after resting, significantly outperforming Bi‐BE. This enhancement can be attributed to the Donnan‐effect‐mimetic IHP, where PO_4_^3−^ adsorption generates a Donnan potential that effectively suppresses electron leakage and mitigates parasitic side reactions through the maintenance of a stable electron confinement structure.

Figure 3h,i display comparative SEM analyses of the Bi‐BE and Bi‐BEP electrodes after 200 electrochemical cycles under open‐circuit potential. A striking morphological contrast is observed: the Bi‐BE electrode develops a fully nanostructured surface that is prone to structural collapse and detachment from the substrate, compromising long‐term stability. In contrast, the Bi‐BEP electrode maintains a relatively smooth surface, thanks to the protection provided by PO_4_^3−^. This smoothness helps minimize the detachment of Bi metal during the alloying process, thereby enhancing both structural integrity and cycling stability. Extended SEM and EDS analysis under open‐circuit potential (Figures S11 and S12, Supporting Information) further demonstrates sustained cycling advantages after 3000 cycles. The Bi‐BEP surface retains remarkable topological homogeneity with uniform P element distribution, highlighting the critical role of PO_4_^3−^ in long‐term stabilization. In stark contrast, the Bi‐BE electrode suffers from extensive nanostructuring and structural degradation, leading to rapid capacity fade. This systematic comparison underscores PO_4_^3−^‐adsorbed IHP as a vital interface‐stabilizing mechanism for aqueous metal electrodes.

Despite surface differences, TEM images reveal that Bi transitions from large particles to nanorods in both BE and BEP electrolytes (Figure S13a,b, Supporting Information). HRTEM analysis confirms that at the charging state, Bi converts to the Na_3_Bi alloy phase in both systems (Figure S14a,b, Supporting Information). IFFT analysis further demonstrates comparable crystallinity in Bi‐BE and Bi‐BEP, indicating that bulk phase transformations are electrolyte‐independent. This result is aligning with the electrochemical data.

Figure 3j presents the cycling stability of Bi‐BE and Bi‐BEP electrodes at a current density of 2 A g^−1^ for 3000 cycles, respectively. Both electrodes exhibit an initial capacity increase, attributed to electrochemical activation. The activation process can be clearly observed from the first 150 cycles of the CV curves at a scan rate of 1 mV s^−1^ (Figure S15, Supporting Information). This phenomenon is primarily due to the characteristics of the Bi metal's large single crystal: in the initial stage, the small solid‐liquid contact area results in a low capacity. As reaction time increases, ion channels gradually open, allowing the capacity to be progressively enhanced. The Bi‐BEP electrode starts with 146.2 mAh g^−1^, peaks at 251.1 mAh g^−1^ after 550 cycles, and retains 81% capacity (200.1 mAh g^−1^) after 3000 cycles. In contrast, Bi‐BE begins with a lower initial capacity (112.3 mAh g^−1^), reaches a maximum of 190.0 mAh g^−1^ after 770 cycles, and suffers rapid degradation, retaining only 55% capacity (105.4 mAh g^−1^) at cycle 3000. A Bi//AC full battery assembled with BEP electrolyte demonstrates excellent cycling stability, with an initial capacity of 82.4 mAh g^−1^, retaining 95.1% of its capacity after 1200 cycles at a current density of 2 A g^−1^ (Figure S16, Supporting Information). The results indicate that the Donnan‐effect‐mimetic IHP plays a significant role in enhancing the electrochemical stability of Bi metal.

To further validate the universal applicability of the Donnan‐effect‐mimetic IHP, we examined its protective effects in two distinct systems: K^+^ storage in Bi metal and Na^+^ storage in BiSn electrodes. For the BiSn‐BEP electrode, the Donnan‐effect‐mimetic IHP enabled exceptional cycling stability, maintaining a remarkable capacity retention of 87.3% after 500 cycles, compared to just 46.3% for the BiSn‐BE electrode (Figure S17, Supporting Information). In the KOH electrolyte system, the PO_4_^3⁻^‐enhanced Bi‐BEP‐K electrode delivered an impressive capacity of 181.5 mAh g^−1^ with a capacity retention of 124.1% after 800 cycles, significantly surpassing the 45.6 mAh g^−1^ of the Bi‐BE‐K electrode without PO_4_^3−^ (Figure S18, Supporting Information). These comparative results unambiguously demonstrate that the Donnan‐effect‐mimetic IHP universally enhances the electrochemical stability of aqueous Bi‐based systems. Compared to some previously reported alkaline Bi‐based electrode materials^[^ ^28^ ^]^ (listed in Table S1, Supporting Information), our Bi‐BEP demonstrates significant advantages in terms of stability, rate capability, and capacity, owing to the construction of the Donnan‐effect‐mimetic IHP.

The superiority is attributed to Donnan‐effect‐mimetic mechanisms through three key functions: i) Ion‐sieving and reaction‐sieving: The Donnan‐effect‐mimetic IHP enables selective Na⁺ permeation while electrostatically repelling OH⁻ ions. This dual functionality enhances electrochemical alloying/dealloying reactions while effectively suppressing parasitic corrosion reactions; ii) The PO₄^3^⁻‐adsorbed IHP establishes a Donnan potential barrier, restricting electron leakage from Bi to the electrolyte, thereby significantly mitigating self‐discharge and corrosion issues; iii) Structural integrity: The PO₄^3^⁻‐adsorbed IHP helps maintain surface stability, preventing the pulverization and detachment of active materials during prolonged cycling.

### 2.4 Electron Management Capability for Donnan‐Effect‐Mimetic IHP

We systematically investigate the impact of PO_4_^3−^‐adsorbed IHP modulation on interfacial electron management. This capability plays a crucial role in addressing self‐discharge and corrosion issues.

**Figure 4:**
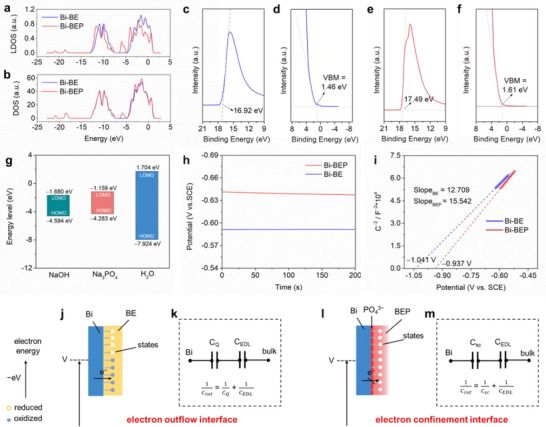
Electronic management capability for Donnan‐effect‐mimetic IHP. a) LDOS and b) DOS spectra for Bi‐BE and Bi‐BEP electrodes. Ultraviolet photoelectron spectroscopy (UPS) of work function for Bi electrode at open‐circuit potential in c) BE and e) BEP; UPS of valence band maximum (VBM) for Bi electrode at open‐circuit potential in d) BE and f) BEP. g) Frontier Molecular Orbital (FMO) analysis for NaOH, Na_3_PO_4,_ and H_2_O. h) Open circuit potential for the Bi electrode in the BE and BEP. i) Mott–Schottky (M–S) plots for the Bi electrode based on electrolytes of BE and BEP. Diagrammatic sketch for j) electron outflow interface of Bi‐BE electrode and l) electron confinement interface of Bi‐BEP electrode; Schematic circuit diagram for k) Bi‐BE interface and m) Bi‐BEP interface.

As shown in **Figures** **4**a,b and S19 (Supporting Information), the density of state (DOS) distributions of Bi‐BE and Bi‐BEP are characteristic of conductive materials. Comparing the two sets of DOS, it is observed that the original DOS peaks have slightly increased in intensity, and new peaks have emerged at ≈−5 to −6 eV and near −20 eV after the adsorption of PO_4_^3−^. The increased valence band DOS peaks indicate that there are more localized electron states as the formation of Bi‐O bonds at the solid‐liquid interface for the Bi‐BEP electrode. The enhanced DOS near the Fermi level suggests greater electron availability for charge transfer. Partial density of states (PDOS) analysis (Figure S20, Supporting Information) reveals that −20 eV peaks stem from PO_4_^3−^ interacting with Bi's s‐orbitals, while the −5 to −6 eV peaks originate from Bi's p‐orbitals. The Donnan effect, arising from immobilized PO_4_^3−^ in the IHP, strengthens these orbital interactions by creating a localized high‐field environment. Local density of states (LDOS) analysis further shows a negative valence band shift for surface Bi atoms, implying reduced energy for electron occupation. This shift is consistent with the Donnan potential counteracting intrinsic band bending, enhancing charge carrier mobility, and surface electron activity.

UPS spectra reveal work functions of 4.3 eV for Bi‐BE and 3.73 eV for Bi‐BEP, respectively (Figure 4c,e). The upward Fermi energy shift for Bi‐BEP aligns with DOS trends and reflects Donnan‐mediated interfacial polarization, where PO_4_^3−^ adsorption induces a surface charge reorganization. The Valence Band Maximum (VBM) position increases from 1.46 eV for Bi‐BE (Figure 4d) to 1.61 eV for Bi‐BEP (Figure 4f), indicating enhanced oxidation resistance after the construction of PO_4_^3−^‐adsorbed IHP. This is because of the Donnan effect, which stabilizes surface charges and mitigates oxidative electron loss, thereby reducing the risk of corrosion.

The addition of Na_3_PO_4_ can also influence the energy levels of a NaOH solution. Na_3_PO_4_ raises the lowest occupied molecular orbital (LUMO) energy level of the BEP electrolyte (−1.680 eV) compared to pure NaOH (−1.159 eV) in Figure 4g, improving electrochemical stability. This result implies that the Donnan effect further modulates ion activity near the interface, elevating the LUMO level by restricting electrolyte reduction through selective PO_4_^3−^ accumulation within the IHP.

Open circuit potentials (OCP) of Bi in BE (−0.591 V) and BEP (−0.638 V versus SCE) in Figure 4h reflect differing surface charge equilibria. The more negative OCP for Bi‐BEP arises from Donnan potential‐driven ion asymmetry, reducing interfacial electron leakage. Mott–Schottky analysis (Figure 4i) confirms a positive flat band potential shift (−0.973 V for BEP versus −1.041 V for BE), indicating reduced electron outflow due to PO_4_^3−^‐adsorbed IHP.^[^ ^29^ ^]^ Bi metal exhibits two different slopes in BE and BEP, primarily due to the varying residual charges on the surface of the Bi metal, resulting in different capacitances.

We can illustrate the two types of solid‐liquid interfaces of the Bi electrode in BE and BEP electrolytes using the schematic circuit diagrams. For the Bi‐BE electrode, the Bi metal surface characteristics contribute to the interface capacitance, which is composed of both quantum capacitance (C_Q_) and double‐layer capacitance (C_EDL_). Electrons accumulated on the Bi‐BE electrode surface can easily leak into the electrolyte, resulting in an electron outflow effect that leads to self‐discharge (Figure 4j,k). In Bi‐BEP, PO_4_^3−^ adsorption alters the electrode surface state, leading to interface capacitance that includes space charge capacitance (C_sc_) via the Donnan effect, confining electrons at the surface while maintaining their activity. This dual mechanism restricts electron flow into the electrolyte while maintaining the activity of electrons on the Bi metal surface, demonstrating an electron confinement effect (Figure 4l,m).^[^ ^30^ ^]^

Therefore, the PO_4_^3−^‐adsorbed IHP establishes electron confinement through the formation of a Donnan potential barrier. This operates through two complementary mechanisms: i) enhancing the surface electron activity of the Bi metal, thereby facilitating charge transfer dynamics during the (de)alloying reaction, and ii) preventing electron outflow from Bi to the electrolyte, thus inhibiting the corrosion reaction, which in turn suppresses self‐discharge and enhances stability.

### 2.5 Ion‐Sieving Effect of the Donnan‐Effect‐Mimetic IHP

**Figure 5:**
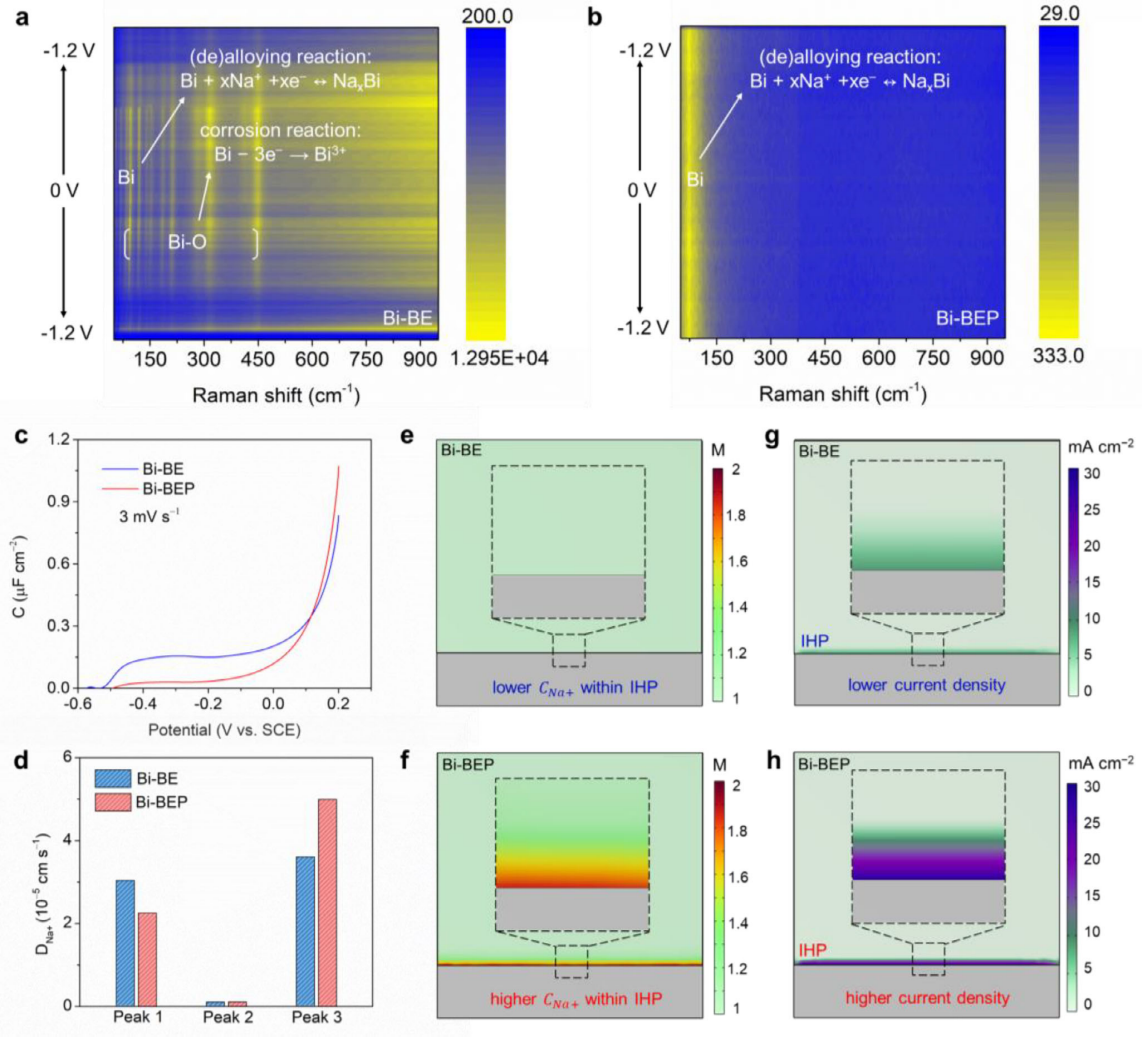
Ion‐sieving effect of the Donnan‐effect‐mimetic IHP. *In‐site* Raman spectra for a) Bi‐BE electrode and b) BEP electrode; c) The non‐Faradaic capacitance‐potential curves for the Bi electrode between 0.2 V and −0.6 V (versus SCE) in BE and BEP electrolytes; d) Calculated D_Na+_ values according to the Randles–Sevcik Equation; Na^+^ ion concentration (C_Na+_) distribution simulation e) Bi‐BE and f) Bi‐BEP interface; Current density simulation at g) Bi‐BE and h) Bi‐BEP interface.

To elucidate the reaction‐sieving effects enabled by the Donnan‐effect‐mimetic IHP on the Bi‐BEP electrode, we conducted in situ Raman spectroscopy to track dynamic phase transitions during charging and discharging processes. For the Bi‐BE electrode (**Figure** **5**a), two characteristic Raman peaks of metallic Bi were observed at 68.6 cm^−1^ (in‐plane E_g_ vibration) and 94.5 cm⁻¹ (out‐of‐plane A_1g_ vibration).^[^ ^31^ ^]^ However, during cycling, these peaks split into shoulder peaks at 55.1 (E_g_) and 85.0 cm^−1^ (A_1g_), indicative of structural asymmetry caused by stress accumulation during Na⁺ alloying (Bi⁺ + xNa⁺ + xe⁻ ↔ Na_x_Bi). Critically, additional peaks emerged in the 100–500 cm^−1^ range: vibrations at 100–200 cm^−1^ correspond to Bi^3+^ motion in [BiO_8_] configurations, while bands at 200–500 cm^−1^ arise from Bi‐O‐Bi, Bi‐O, and Bi‐O⁻ vibrations,^[^ ^32^ ^]^ unambiguously confirming the formation of Bi_2_O_3_ corrosion products via the parasitic reaction (Bi − 3e^−^ →Bi^3+^). This corrosion is driven by the unregulated presence of H_2_O and OH⁻ within the IHP, which facilitates interfacial ion exchange and destabilizes the Bi‐electrolyte interface.

In stark contrast, the Bi‐BEP electrode (Figure 5b) with PO_4_^3−^‐adsorbed IHP exhibits no detectable Bi_2_O_3_ signals across 100–1000 cm^−1^, demonstrating that the Donnan‐effect‐mimetic IHP effectively isolates Bi from corrosive species. The recovery of the E_g_ (68.6 cm^−1^) and A_1g_ (94.5 cm^−1^) peak symmetry further reveals reduced structural stress within the Bi matrix.^[^ ^27^ ^]^ These results suggest that the Bi‐BEP with Donnan‐effect‐mimetic IHP enables reaction‐sieving and enhances the reversibility of the electrochemical alloying and dealloying reactions by facilitating ion selectivity: it electrostatically excludes OH⁻ through charge‐selective Donnan partitioning, while permitting Na⁺ transport for alloying reaction.

Linear sweep voltammetry (LSV, Figure S21, Supporting Information) further reveals non‐Faradaic processes (0.2 to −0.6 V versus SCE), while AC voltammetry (Figure 5c) shows a shifted potential of zero charge (PZC) for Bi‐BEP, reflecting PO_4_^3−^‐induced restructuring of adsorbed species within the IHP. Kinetic enhancement via Donnan effect ion partitioning was quantified through Na⁺ diffusion coefficients (*D*_Na⁺_). Using the Randles–Sevcik Equation 3:

(3) $$i_{p}={\mathrm{kn}}^{3/2}{\mathrm{AD}}^{1/2}{\mathrm{cv}}^{1/2}$$

where *n, A, C, D*, and *v* represent electron transfer number, area of Bi electrode, ion concentration, ion diffusion coefficient, and scan rate, respectively, from Figure S22a,b (Supporting Information).^[^ ^33^ ^]^ The current maximum value (*i_p_*) was proportional to the scan rate (*v^1/2^*), the slope is *kn^3/2^AD^1/2^c*. The calculated D_Na+_ values of the Bi‐BEP electrode of the alloying reaction (P3) and the first‐step (P2) and second‐step (P1) dealloying reaction were 5.00 × 10^−5^, 1.09 × 10^−6,^ and 2.26 × 10^−5^, respectively. Whereas, the corresponding D_Na+_ values for the Bi‐BE electrode were 3.59 × 10^−5^, 8.65 × 10^−7,^ and 3.02 × 10^−5^, respectively (Figure 5d). This kinetic enhancement stems from the Donnan‐driven Na⁺ concentration gradient: the PO₄^3^⁻‐adsorbed IHP electrostatically attracts and localizes Na⁺ at the electrode interface, creating an ion‐rich zone that accelerates alloying dynamics.

Finite element analysis (FEA) was further employed, integrating a theoretical visualization model grounded in experimental data. The model architecture incorporates experimentally derived ionic compositions, concentration gradients, and electrode configuration, enabling precise reconstruction of Na⁺ spatial distribution maps and interfacial current density patterns at the solid‐liquid interface. A representative unit cell was constructed, comprising the Bi electrode and electrolyte domain, with boundary isolation implemented via insulating edge strips to eliminate peripheral interference.

The composition of chemical species at the interface determines the reaction pathway selection between alloying and corrosion mechanisms, while the localized ionic concentration governs the kinetics of electrochemical processes. As evidenced by our simulations, PO_4_^3−^ adsorption at the electrode‐electrolyte interface for Bi‐BEP significantly modulates the distribution of reactive ions and interfacial current density via Donnan partitioning. For Bi‐BE (Figure 5e), Na⁺ distributes homogeneously and at a lower current density (Figure 5g) due to competition with OH⁻/H_2_O at the unregulated interface. For Bi‐BEP (Figure 5f), Donnan electrostatic forces concentrate Na⁺ at the interface compared to Bi‐BE, forming a charge‐selective ion reservoir that amplifies interfacial current density (Figure 5h).

This Donnan‐driven ion redistribution directly modulates reaction pathways, as quantified by the *Butler‐Volmer* Equation 4:^[^ ^30^, ^34^ ^]^

(4) $$i=i_{0}\;\left(\exp\left(\frac{\alpha_{a}F\eta }{RT}\right)-\;\exp\left(\frac{-\alpha_{c}F\eta }{RT}\right)\right)$$

where *i* represents the electrode current density, *i*_0_ is the exchange current density, α_*a*_ and α_*c*_ are the dimensionless charge transfer coefficients, *F* is the Faraday constant, η is activation overpotential, *R* is the universal gas constant, and *T* is temperature. In Bi‐BEP, the Na⁺‐enriched interface elevates *i₀* (versus Bi‐BE), shifting the equilibrium toward the alloying reaction (Bi ↔ Na_x_Bi) and away from the corrosion reaction. Conversely, Bi‐BE exhibits dual competing currents (*i_alloying_* and *i_corrosion_*), reducing interfacial current density and destabilizing cycling.

This reaction‐sieving mechanism, enabled by the Donnan‐effect‐mimetic IHP, ensures preferential Na⁺ access to the Bi surface while blocking reactive OH⁻, thereby decoupling the desired alloying process from parasitic corrosion. The absence of side product of Bi_2_O_3_ signatures in the Bi‐BEP highlights the IHP's ability to enforce interfacial ion specificity, a key feature of Donnan exclusion. As a result, the Bi‐BEP electrode demonstrates enhanced structural and chemical stability in aqueous electrolytes, highlighting the crucial role of Donnan‐mimetic IHP in suppressing side reactions and enabling ion‐sieving and reaction‐sieving electrochemistry. This, in turn, leads to the realization of ultra‐stable cycling stability and exceptional capacity output.

## 3 Conclusion

In conclusion, we innovatively extend the classical Donnan model to dynamic electrochemistry to stabilize the solid‐liquid interface of aqueous metal electrodes. Using Bi as a model platform, we engineered a Donnan‐effect‐mimetic IHP through strong PO_4_^3−^ chemisorption, creating a stable Donnan equilibrium at the electrode‐electrolyte interface. This engineered interface enables three key functions: i) ion and reaction sieving through charge‐selective ion partitioning, enriching Na⁺ while excluding OH⁻, thereby facilitating selective (de)alloying over corrosion; ii) electron confinement via the Donnan potential to minimize parasitic electron leakage; and iii) dynamic stabilization of the IHP through strong anion chemisorption, integrating the classical Donnan model with practical electrochemical cycling. Leveraging the Donnan‐effect‐mimetic IHP, the Bi electrode demonstrates exceptional cycling stability, maintaining 200 mAh g^−1^ (81% capacity retention) after 3000 cycles at 2 A g^−1^, and superior capacity (134 mAh g^−1^ at 16 A g^−1^). Our research establishes a universal interfacial paradigm through the extension of the classical Donnan effect to dynamic IHP engineering. The demonstrated synergy of charge‐selective ion sieving and electron confinement provides a fundamental solution to decouple desired electrochemical processes from parasitic reactions, offering a transformative platform for next‐generation RAMBs and beyond.

## Conflict of Interest

The authors declare no conflict of interest.

## Supporting information



Supporting Information

## Data Availability

The data that support the findings of this study are available in the supplementary material of this article.
