# Supplementary material for: Donnan‐Engineered Inner Helmholtz Plane Enabling Ultra‐Stable Aqueous Bismuth Electrode
Source: Adv Sci (Weinh). 2025 Jul 29;12(39):e08965. doi: 10.1002/advs.202508965 (PMC12533370; doi:10.1002/advs.202508965)
Supplement: Supplementary file 1 — Supporting Information [file ADVS-12-e08965-s001.doc]

**Supporting Information**

Donnan-Engineered Inner Helmholtz Plane Enabling Ultra-Stable Aqueous Bismuth Electrode

**Experimental**

**Chemicals**

Bismuth nitrate pentahydrate (Bi(NO_3_)_3_·5H_2_O), Tin chloride pentahydrate (SnCl_4_·5H_2_O), sodium hydroxide (NaOH), potassium hydroxide (KOH), trisodium phosphate dodecahydrate (Na_3_PO_4_·12H_2_O), tripotassium phosphate dodecahydrate (K_3_PO_4_·12H_2_O), ethylene glycol (C_2_H_6_O_2_), Activated carbon (AC) were obtained from Chemical Reagents Limited Company. Nafion, nickel (Ni) foam and conducting graphite were purchased from Aladdin.

**Synthesis of** **Bi electrodes**

Bi metal electrodes are fabricated using a simple redox method (Bi^3+^ + Ni → Ni^2+^ + Bi^0^), where nickel (Ni) foam serves both as a reducing agent and a substrate^[1]^ The detailed procedural steps are as follows:

Before the synthesis of Bi electrode, nickel foam was cut into rectangles (1 × 1.5 cm^2^; thickness, 1 mm). It was then carefully washed with 0.2 M hydrochloric acid, followed by deionized water and ethanol to remove the passive layer.

In a typical synthesis of the Bi electrode, Bi(NO₃)₃·5H₂O (0.2 mmol) was dissolved in 2 mL of C_2_H_6_O_2_ solution in a glass bottle within an argon-filled glove box. The clean Ni foam (1 × 1.5 cm²) was then immersed in the solution. The glass bottle was tightly sealed and maintained at 60 °C. After 15 hours, Bi particles formed on the surface of the Ni substrate. The substrate was then removed, washed with deionized water, and dried in a vacuum at 60 °C for 10 hours.

The quality of the active substance Bi metal was measured by Inductively Coupled Plasma Spectrometer (ICP).

**Synthesis of** **BiSn electrode**

Dissolve Bi(NO_3_)_3_·5H_2_O and SnCl_4_·5H_2_O in a 10:1 ratio in 50 mL of 0.5 M HCl solution, and stir for 20 minutes until completely dissolved. The BiSn electrode was prepared *via* an electrodeposition process: A carbon fiber cloth (1 × 1.5 cm^2^) is used as the working electrode, a saturated Ag/AgCl electrode as the reference electrode, and a platinum sheet as the counter electrode. A constant current density of −25 mA·cm^−2^ is applied in the prepared electrolyte for 400 seconds to deposit the BiSn electrode material. After electrodeposition, the electrodes are washed multiple times with distilled water and ethanol, followed by drying in a vacuum oven at 60 °C for 6 hours.

**Synthesis of BE,** **BEP, BE-K and** **BEP-K electrolytes**

An aqueous solution of NaOH (1 M) or KOH (1 M) is referred to as the baseline electrolyte (BE) or BE-K, respectively. The BE or BE-K, which include the addition of Na_3_PO_4_·12H_2_O or K_3_PO_4_·12H_2_O, are referred to as BEP or BEP-K, respectively. BEP and BEP-K electrolytes were prepared by mixing BE with Na_3_PO_4_·12H_2_O solution (0.333 M) and BE-K with K_3_PO_4_·12H_2_O solution (0.333 M), respectively, in a 25 : 1 volume ratio. For testing, each electrolytic cell was filled with 25 mL of electrolyte.

**Synthesis of AC electrode**

AC electrodes were prepared using a coating method. AC powder, conductive graphite, and Nafion were mixed in a mass ratio of 8:1:1. The mixture was stirred with a magnetic bar for 12 hours to form a homogeneous slurry. The slurry was then coated onto carbon fiber paper (1 x 1.5 cm^2^) and dried in an oven at 60 °C for 12 hours. Then, the dried AC electrodes can be reserved for use as the cathodes.

**Physicochemical Characterizations**

X-ray diffraction (XRD) patterns were obtained using a D8 Advance X-ray diffractometer (Bruker, Germany) with Cu Kα radiation (λ = 1.5406 Å). Scanning electron microscopy (SEM, Hitachi S-8010) and transmission electron microscopy (TEM, JEM-2100F) were performed for a comprehensive analysis of the samples' morphology and structure. Raman spectra were collected using a LabRAM ARAMIS micro-Raman spectrometer (Horiba-Jobin Yvon, Germany) with a cobalt laser (532 nm) and a 1% filter. X-ray photoelectron spectroscopy (XPS) analysis was conducted with a photoelectron spectrometer (Kratos Axis Supra, Shimadzu, Japan), using a standard binding energy of C1s at 284.8 eV. Time of Flight Secondary Ion Mass Spectrometry (TOF-SIMS) was performed using TESCAN S9000G.

**Electrochemical Characterizations**

The electrochemical performance of a single Bi electrode was evaluated through a conventional three-electrode system: the Bi electrode, Pt foil and saturated calomel electrode (SCE) were used as counter and reference electrodes, respectively. Cyclic voltammetry (CV), galvanostatic charge-discharge (GCD), GCD cycling test, and AC impedance spectroscopy measurements were conducted on an electrochemical workstation (CHI660E) and Neware testing system. All electrochemical measurements were conducted in ambient conditions.

**Interfacial Exchange Current Density**

Interfacial exchange current density at the interface is a crucial parameter for evaluating electrochemical reactions at the solid-liquid interface. It can be quantified using the extended *Nernst-Planck* equation:^[2]^


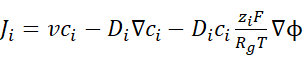
 (S1)

At the interface, mass exchange primarily occurs through electrochemical reactions and ion migration, involving electrical migration, diffusion, and convection. Considering that the influence of convection is minimal and can be disregarded at the interface, the equation involves terms for the velocity of the fluid (
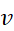
), concentration of the ith component (
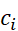
), diffusion coefficient of the ith component (
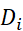
), valence of the ith component (
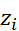
), Faraday constant (
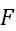
), gas constant (
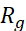
), absolute temperature (
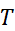
), and electric potential (
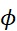
). The first term (
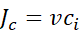
) is induced by convection (which is neglected). The second term (
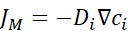
) is produced by the migration due to concentration gradients, and the third term (
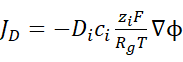
) is generated by the movement of charged species influenced by electric potential gradients. For metal electrodes, the current density at the interface is primarily influenced by ion migration and diffusion processes, which reflects the electrochemical reaction activity.

**Calculation of flat band potential**

The Bi electrode exhibits different solid-liquid interfaces due to the variation in surface residual charges between BE and BEP electrolytes. This discrepancy leads to an offset in the flat band potential and influences the extent of band bending at the solid-liquid interface. The flat band potential can be obtained basing on the *Mott-Schottky* Equation S2:^[3]^


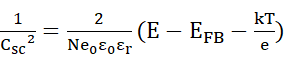
 (S2)


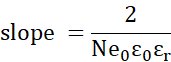
 (S3)

In the equation, ε represents the relative dielectric constant, ε_0_ is the vacuum dielectric constant, N is the donor or acceptor density; E and E_fb_ denote the electrode potential and flat-band potential, respectively. The intercept of the extension of the *Mott-Schottky* plot on the voltage axis provides E_fb_.

**Calculation of the *b* values**

The potential-dependent current responses of an electrochemical process of an electrode come from two types of contributions *i.e.*, semi-infinite diffusion-controlled and surface-dominated capacitive processes. The diffusion-controlled processes are Faradaic processes which are controlled by the diffusions of electroactive species in bulk electrode materials. The fast capacitive contributions stem from the electric double layer capacitor and Faradaic processes on the (sub)surface of electrode materials. The relationship between current density (*i*) and scan rate (*v*) can be used to describe charge transfer control factors. The relationship from a cyclic voltammogram (CV) obeys the Equation (S4):

*log(i) = blog(v) + log(a)* (S4)

Where *b* represents the slope of *log(i)* vs. *log(v)* curve. Whereas, *b =* 0.5 indicates a semi-infinite diffusion-controlled process and *b =* 1 indicates an ideal surface-dominated capacitive process.^[1, 4]^

**Calculation of specific capacity of single electrodes and full cells**

Specific capacity (*C*) of single electrode can be calculated by GCD curves using the Equation (S5):


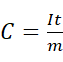
 (S5)

Where *C* (mAh g^−1^) is the mass specific capacity, *I* (mA) is the constant discharging current, Δ*t* (h) is the discharging time, *m* (g) is the mass of active substance.

Device capacity (*C*_cell_) of a full cell can be calculated through GCD curves using the Equation (S6):


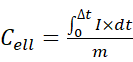
 (S6)

Where *C*_cell_ (mAh g^−1^) is the areal capacity, *I* (mA) is the discharging current, Δ*t* (h) is the discharging time and *m* (g) is the mass of active substance.

**Computational details**

**Calculations of adsorption energies**

The calculations of adsorption energies are performed in the framework of the density functional theory with the projector augmented plane-wave method, as implemented in the Vienna ab initio simulation package.^[5]^ The generalized gradient approximation proposed by Perdew, Burke, and Ernzerhof is selected for the exchange-correlation potential.^[6]^ The long-range *van der Waals* interaction is described by the DFT-D3 approach.^[7]^ The cut-off energy for plane wave is set to 500 eV. The energy criterion is set to 10^−6^ eV in iterative solution of the Kohn-Sham equation. A vacuum layer of 15 Å is added perpendicular to the sheet to avoid artificial interaction between periodic images. The K-mesh resolved in real space is 0.04 2π Å^−1^. All the structures are relaxed until the residual forces on the atoms have declined to less than 0.03 eV Å^−1^.

**Ab initio molecular dynamics (AIMD) simulation**

The Ab initio molecular dynamics (AIMD) simulation was carried out with CP2K package (version 7.1) in the framework of the density functional theory,^[8]^ based on the hybrid Gaussian and plan-wave scheme.^[9]^ The molecular orbitals of the valence electrons were expanded into DZVP-MOLOPT-SR-GTH basis sets,^[10]^ while atomic core electrons are described through Goedecker-Teter-Hutter (GTH) pseudopotentials.^[11]^ A plane-wave density cutoff of 500 Ry was adopted. The long -range *van der Waals* interaction is described by the DFT-D3 approach.^[7]^ During the simulation, the temperature was controlled by CSVR thermostat, and the time step was set to 1 *fs* with the hydrogen atoms replaced by deuterium atoms.

**Binding energy and electron density distribution**

The calculations of the interaction between various ions and Bi (012) substrate are performed in ORCA program with the implicit solvent models^[12]^. The PBE generalized gradient approximation (GGA) methods were utilized to describe the exchange-correlation functions. PBE0-D3(BJ)/def2-SV(P) functional is utilized to optimize the structure. The charge distribution and interactions within the system are simulated by setting an effective dielectric constant of 78.54 to characterize the polarization capability of the water solvent. The binding energy (E_B_) between different ions and Bi (012) substrate is defined according to the following formula:

E_B_ = Es - E_1_ -E_2_

E_S_, E_1_ and E_2_ represent the total energy of ion-solvent system, total energy of unit 1 (ions), total energy of unit 2 (Bi substrate), respectively. In addition, a corresponding charge of ions was assigned to the systems, which equals the charge of the absorbed ion itself.

**The density of states (DOS)**

The first-principles calculations of the DOS of the materials were done by using the plane wave ultrasoft pseudo-potential within the Cambridge sequential total energy package (CASTEP)^[13]^. The PBE generalized gradient approximation (GGA) methods were utilized to describe the exchange-correlation functions. A 2 × 2 × 1 supercell was employed to ensure computational efficiency, and the plane wave function's energy cutoff was set to 400 eV, ensuring a total energy convergence of 1 × 10^−5^ eV atom^−1^. During relaxation, all atoms were adjusted until the force on each ion converged to values not exceeding 0.03 eV Å^−1^, with a stress below 0.05 GPa through the Broyden, Fletcher, Goldfarb, Shanno (BFGS)-based method.

**Figures and Tables**


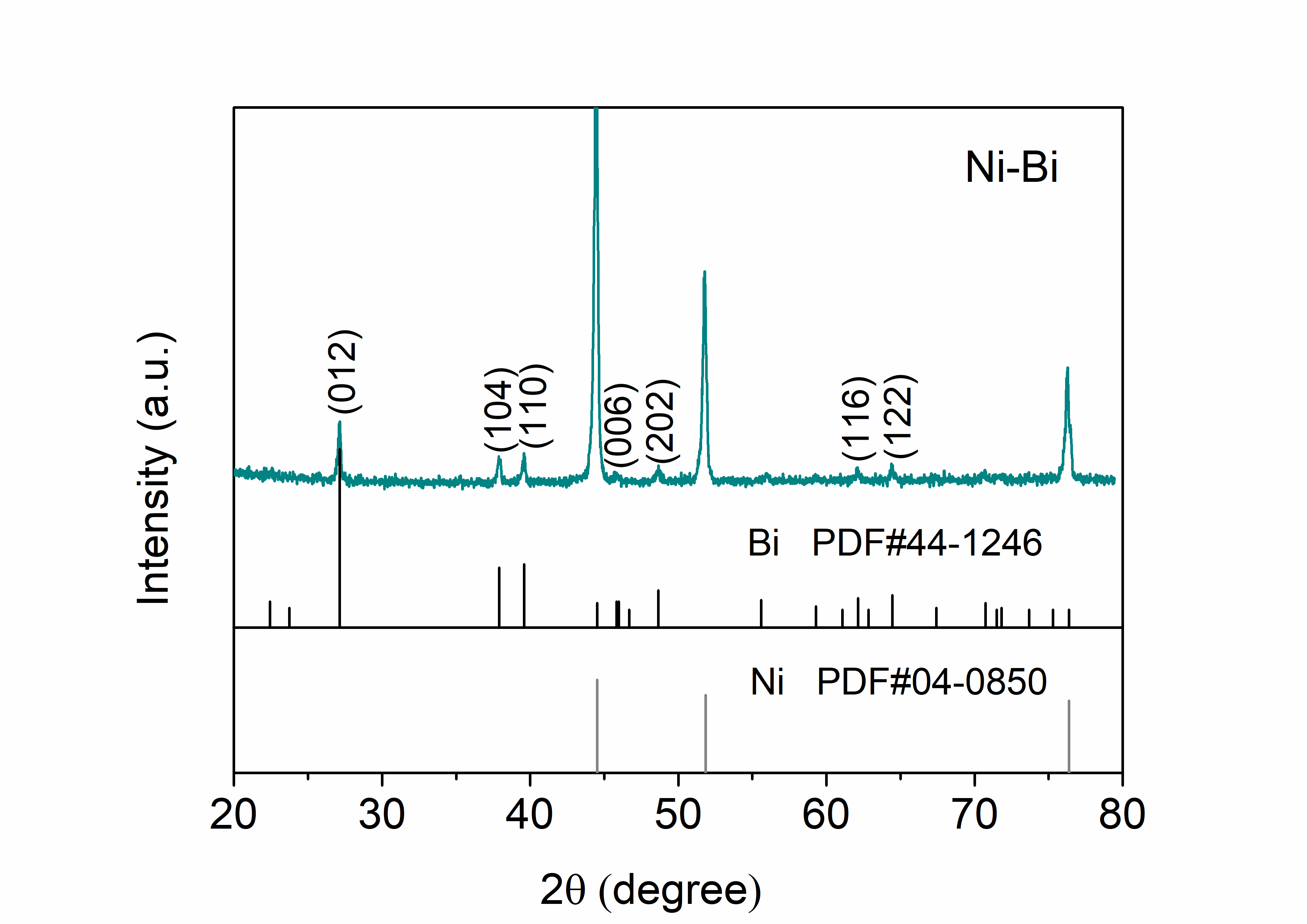


**Figure S1.** XRD patterns for Bi electrode.


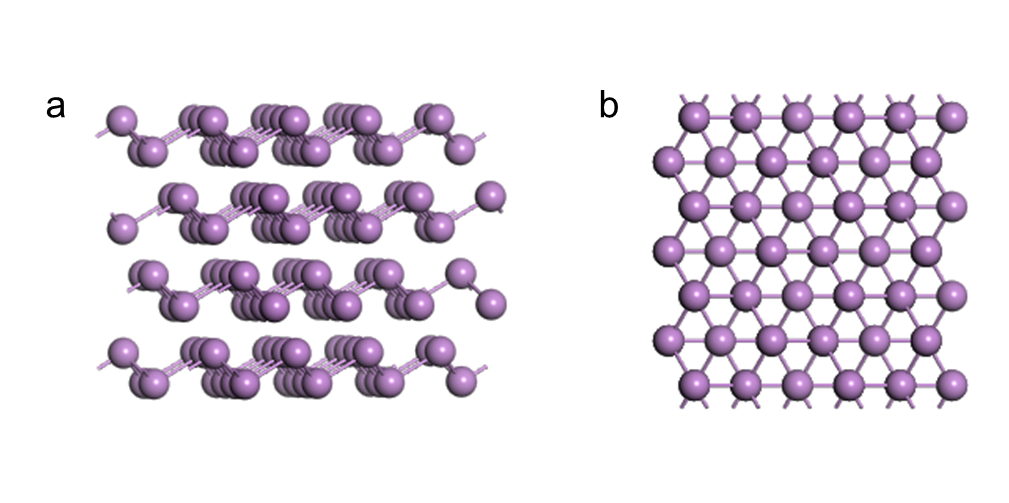


**Figure S2**. Crystal structure of Bi **a)** at side view; **b)** top view.


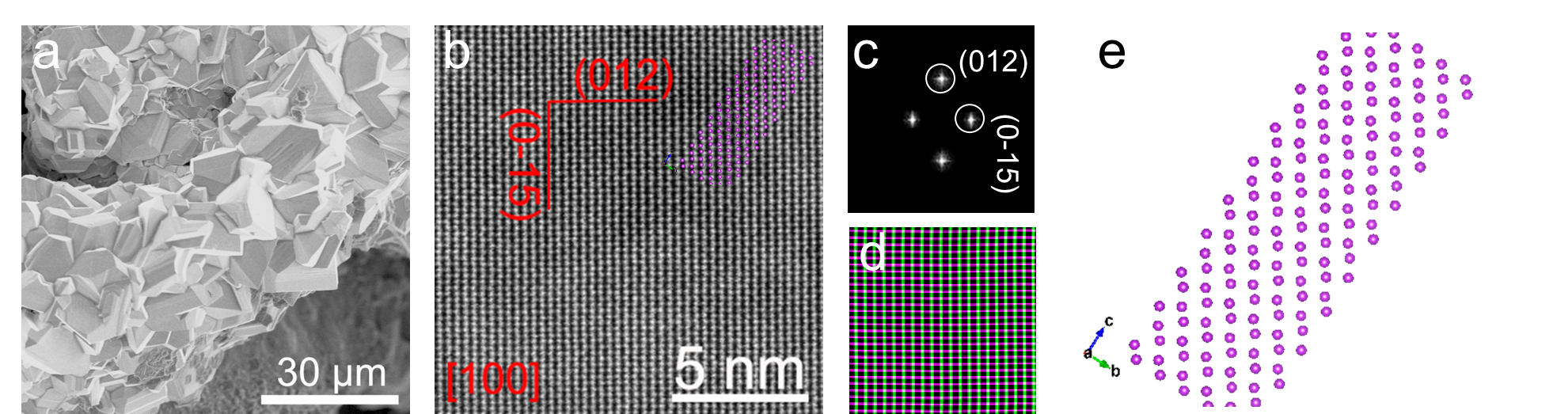


**Figure S3. a)** SEM for Bi single crystal on Ni foam; **b)** HRTEM; **c)** FFT image; **d)** IFFT image; **e)** atomic arrangement along with [100] zone axis.

Bi metal electrodes are prepared using a simple redox method (Bi^3+^ + Ni → Ni^2+^ + Bi^0^), where nickel (Ni) foam serves both as a reducing agent and a substrate (see details in *Experimental*).^[1]^ X-ray diffraction (XRD) patterns (Figure. S1) reveal well-crystallized Bi metal with sharp characteristic diffraction peaks, in alignment with PDF#44-1246, *R-3m*.^[14]^ The crystal structure of Bi presents a graphite-like hexagonal layered arrangement, wherein interlayer connections are enabled by *van der Waals* forces (as depicted in Figure. S2), with an interlayer spacing measuring 0.395 nm. Scanning electron microscope (SEM) image (Figure. S3a) indicates that Bi particles are distributed on the Ni foam, with particle sizes ranging from 3-5 μm. Furthermore, high-resolution transmission electron microscopy (HRTEM) observations reveal that the interplanar spacing measures 0.328 nm and 0.203 nm at a 90° crystal plane angle, corresponding to the (012) and (0-15) planes of metallic Bi (Figure. S3b). These planes align with the [100] crystallographic axis, a finding supported by the Fast Fourier Transform (FFT) analysis in Figure. S3c. The inverse Fast Fourier Transform (IFFT) depicted in Figure. S3d further underscores the high crystallinity of the Bi metal. Additionally, Figure. S3e provides a visual representation of the atomic distribution aligned with the [100] crystallographic axis on the electrode surface.


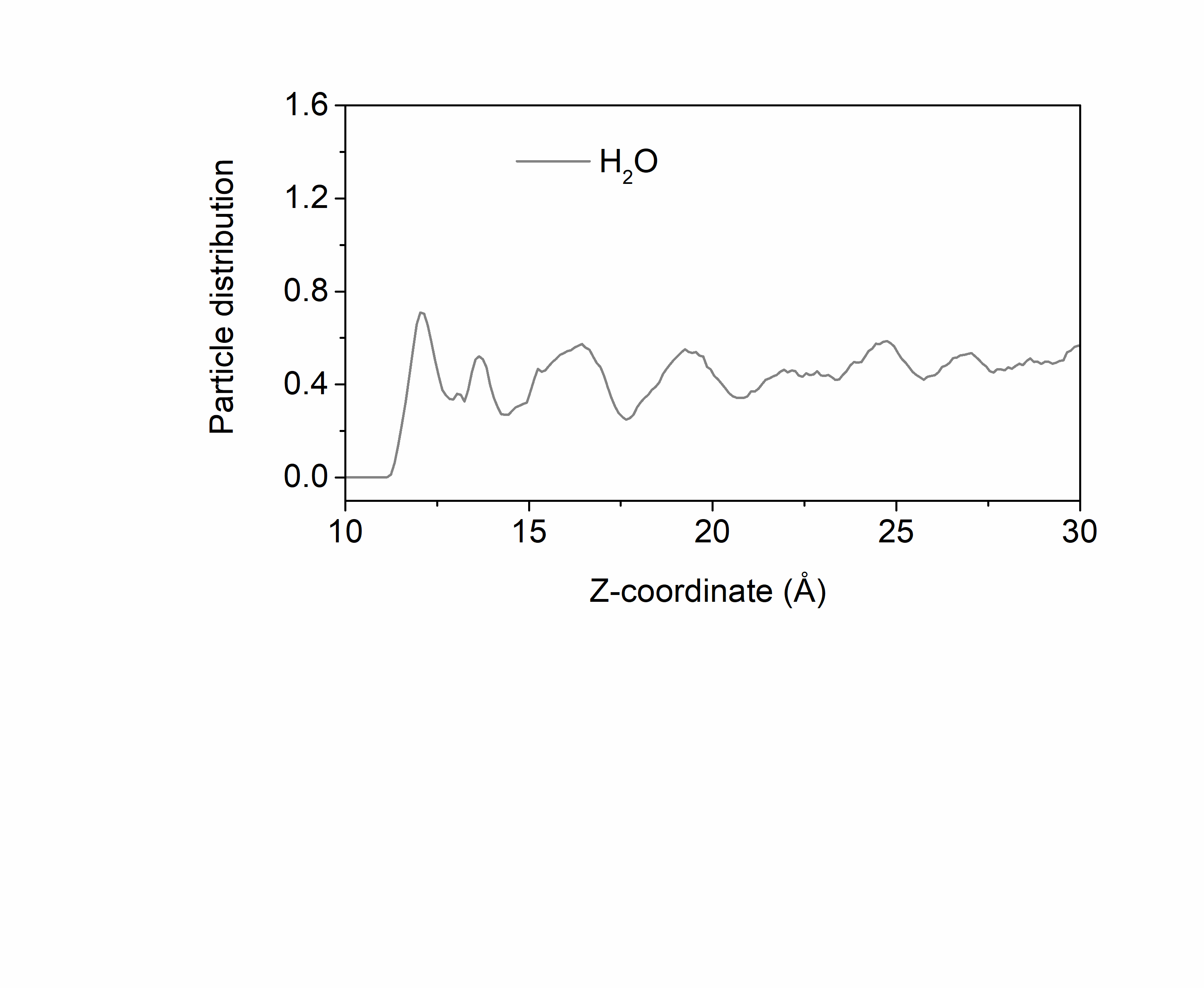


Figure S4. Statistical H_2_O distribution at the Bi(012)/electrolyte interface.


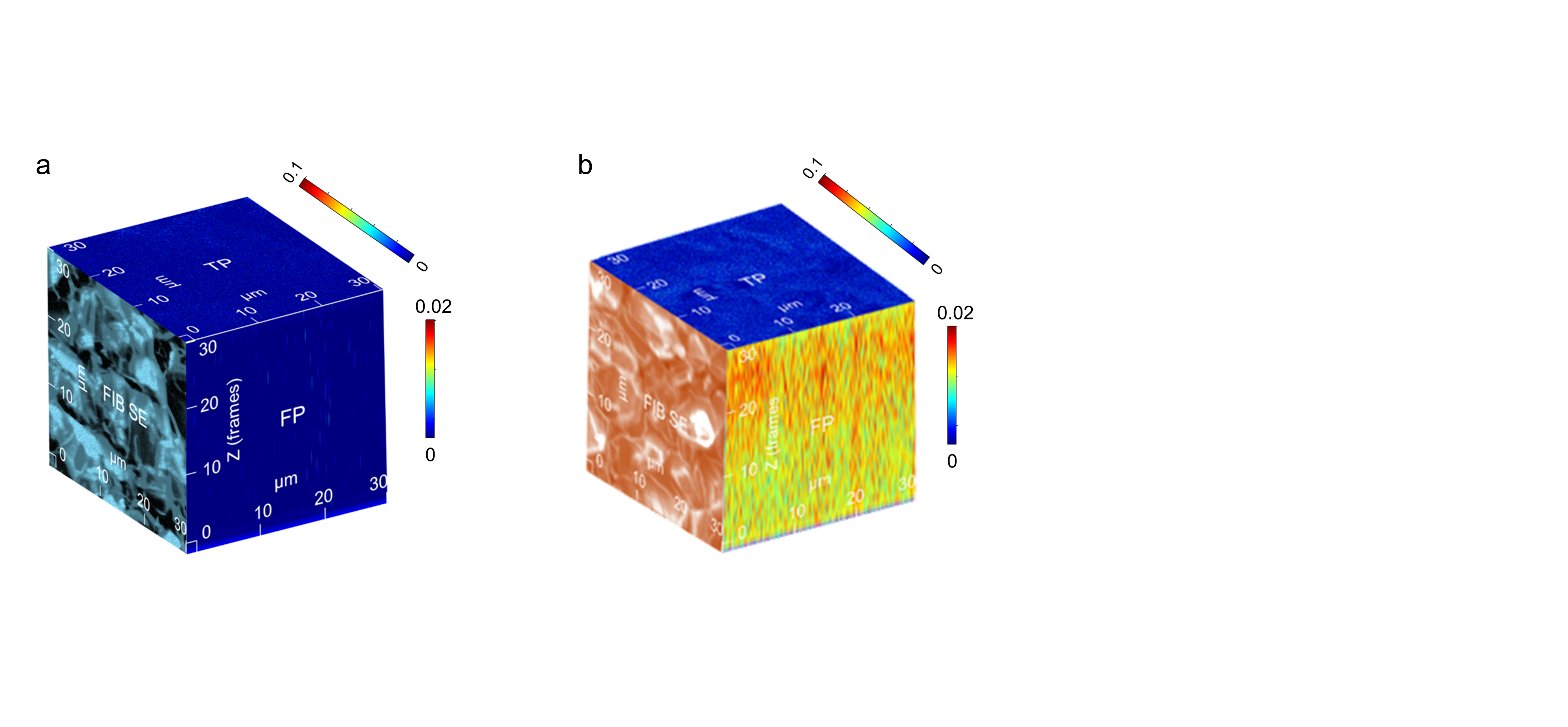


Figure S5. TOF-SIMS pattern of P element on the Bi surface (top projection) and perpendicular to Bi surface (front projection) for Bi-BEP a) before and b) after 5 cycles (at the charging state of −1.2 V vs. SCE).

Figure S6. Statistical content of P element before and after 5 cycles (at the charging state of −1.2 V vs. SCE) in the TOF-SIMS test area.

Time-of-Flight Secondary Ion Mass Spectrometry (TOF-SIMS) was conducted to track the presence of P on the Bi surface (top projection) and perpendicular to the Bi surface (front projection) for Bi-BEP before and after undergoing 5 cycles. The content of P element on the Bi after cycling for 5 cycles (**Figure. S5b**) is significantly larger than that before cycling (**Figure. S5a**). The content of P is nearly eight times higher after 5 cycles of cycling for Bi electrode with BEP electrolyte (**Figure. S6**). This result indicates that P element can adsorb on the surface of Bi along with the electrochemical processes.


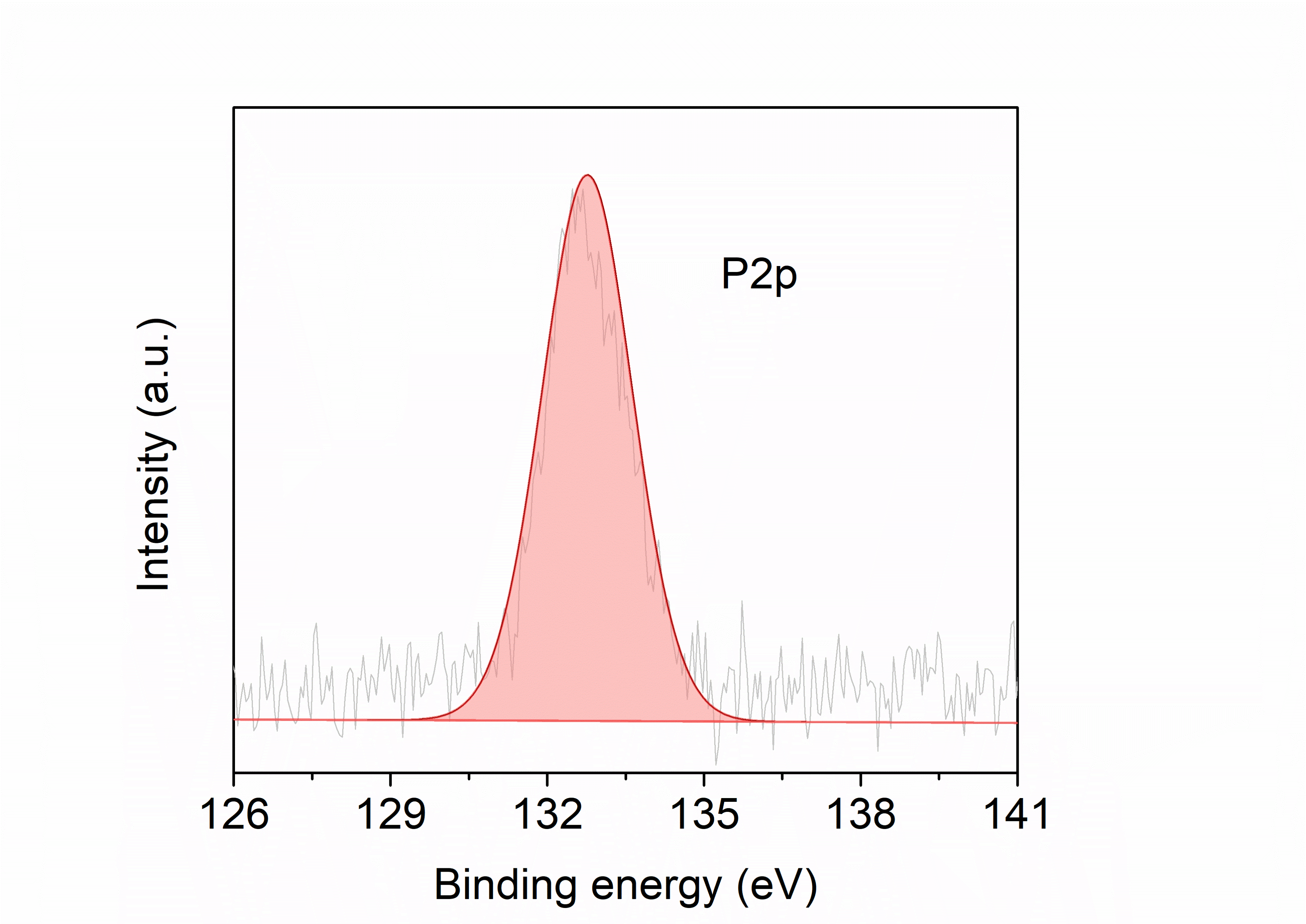


Figure S7. P2p spectra for Bi-BEP electrode (at the charging state of −1.2 V vs. SCE).


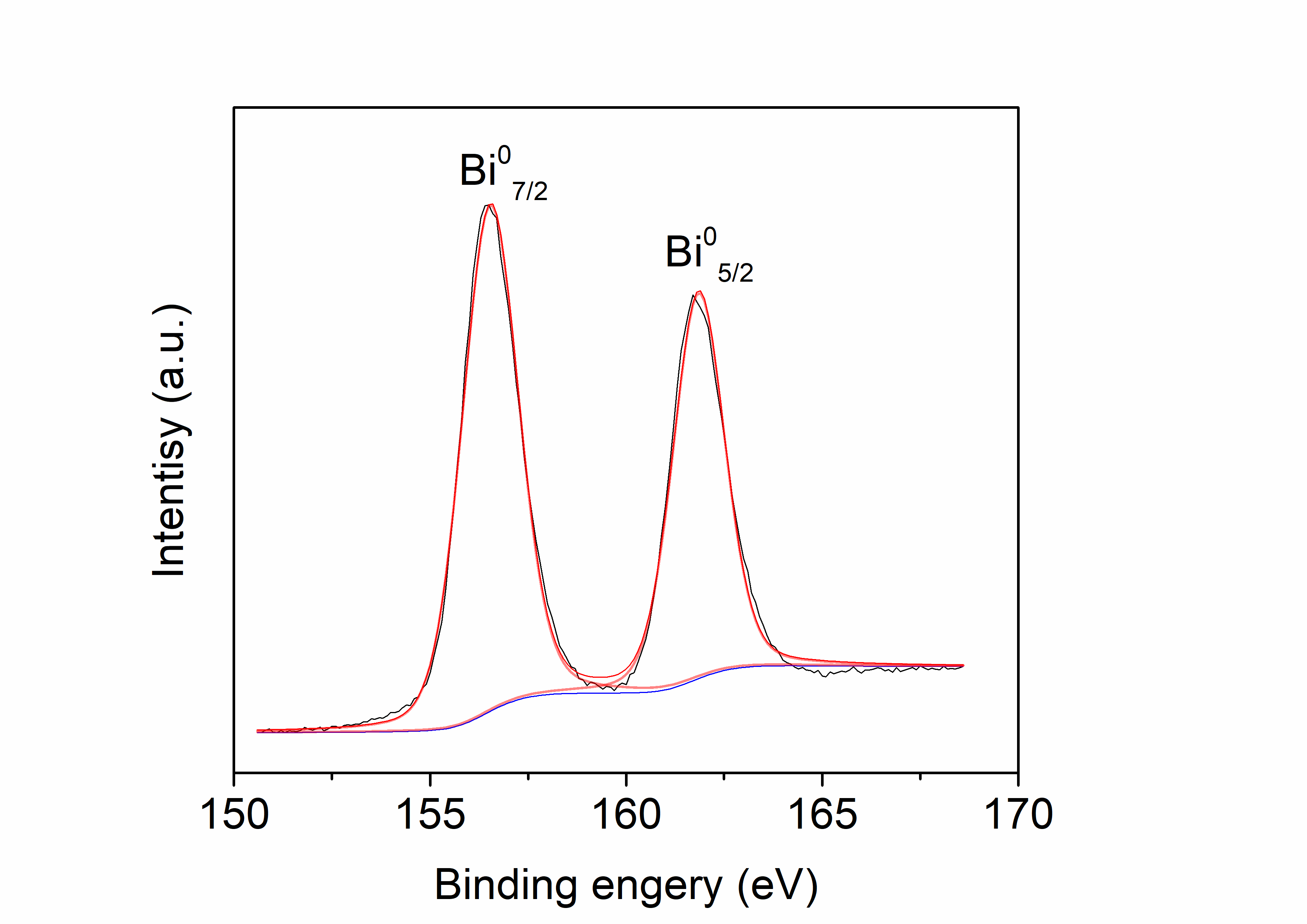


Figure S8. Bi4f XPS spectra for Bi electrode.


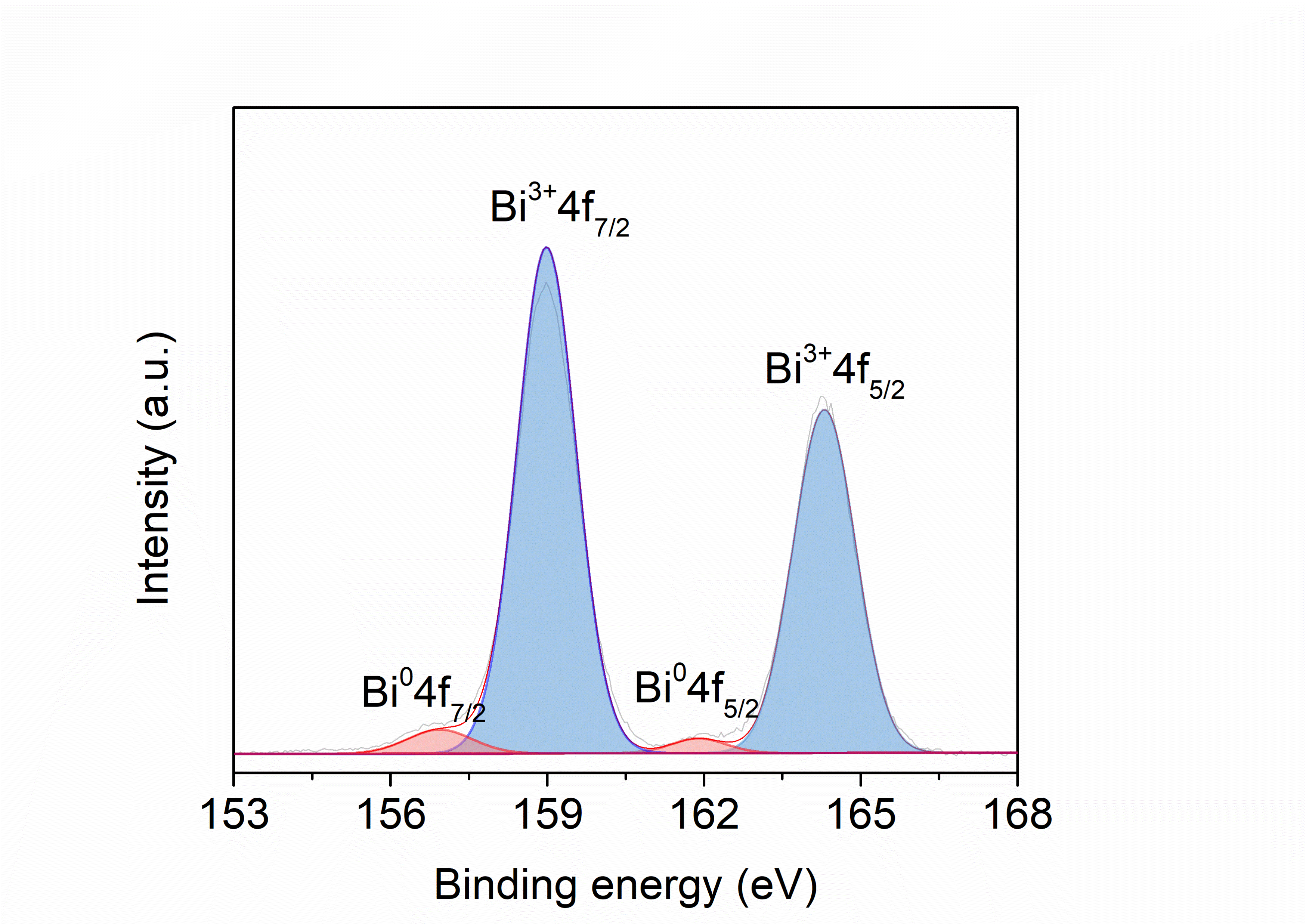


Figure S9. Bi4f XPS spectra for Bi-BEP electrode (at the charging state of −1.2 V *vs.* SCE).

The P2p peak centered at 132.8 eV of PO_4_^3−^ is evident in the P2p XPS spectra (**Figure. S7**), indicating that PO_4_^3−^ can adsorb onto the surface of the Bi metal. The Bi4f XPS analysis of the pure Bi electrode is depicted in **Figure. S8**. Two fitted peaks at binding energies of 156.4 and 161.7 eV correspond to the typical 4f7/2 and 4f5/2 peaks of Bi^0^. In the Bi-BEP electrode, apart from the two Bi^0^ peaks, two remaining peaks centered at 158.16 and 163.46 eV are assigned to Bi^3+^,^[15]^ indicating electron sharing between the Bi metal and PO_4_^3−^, resulting in a positive shift compared to the peaks of Bi^0^ (**Figure. S9**).^[16]^


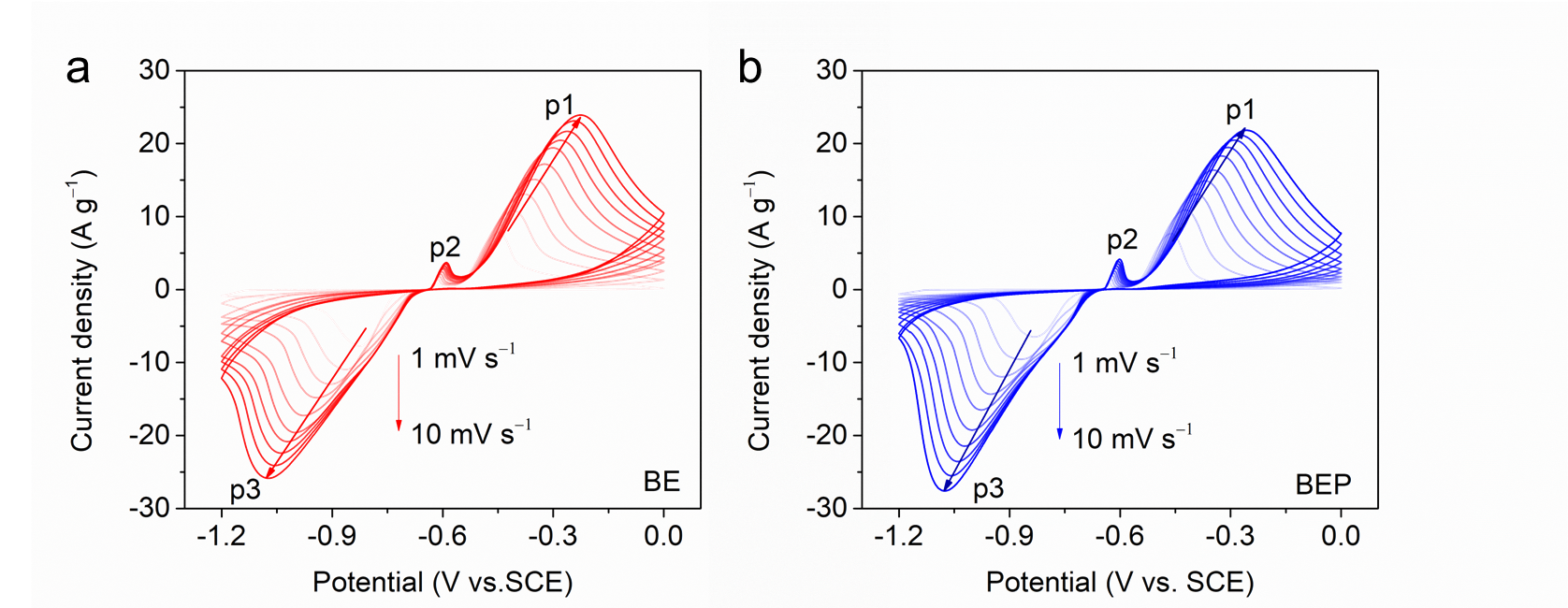


Figure S10. CV curves at different scan rates for a) Bi-BE and b) Bi-BEP electrode.


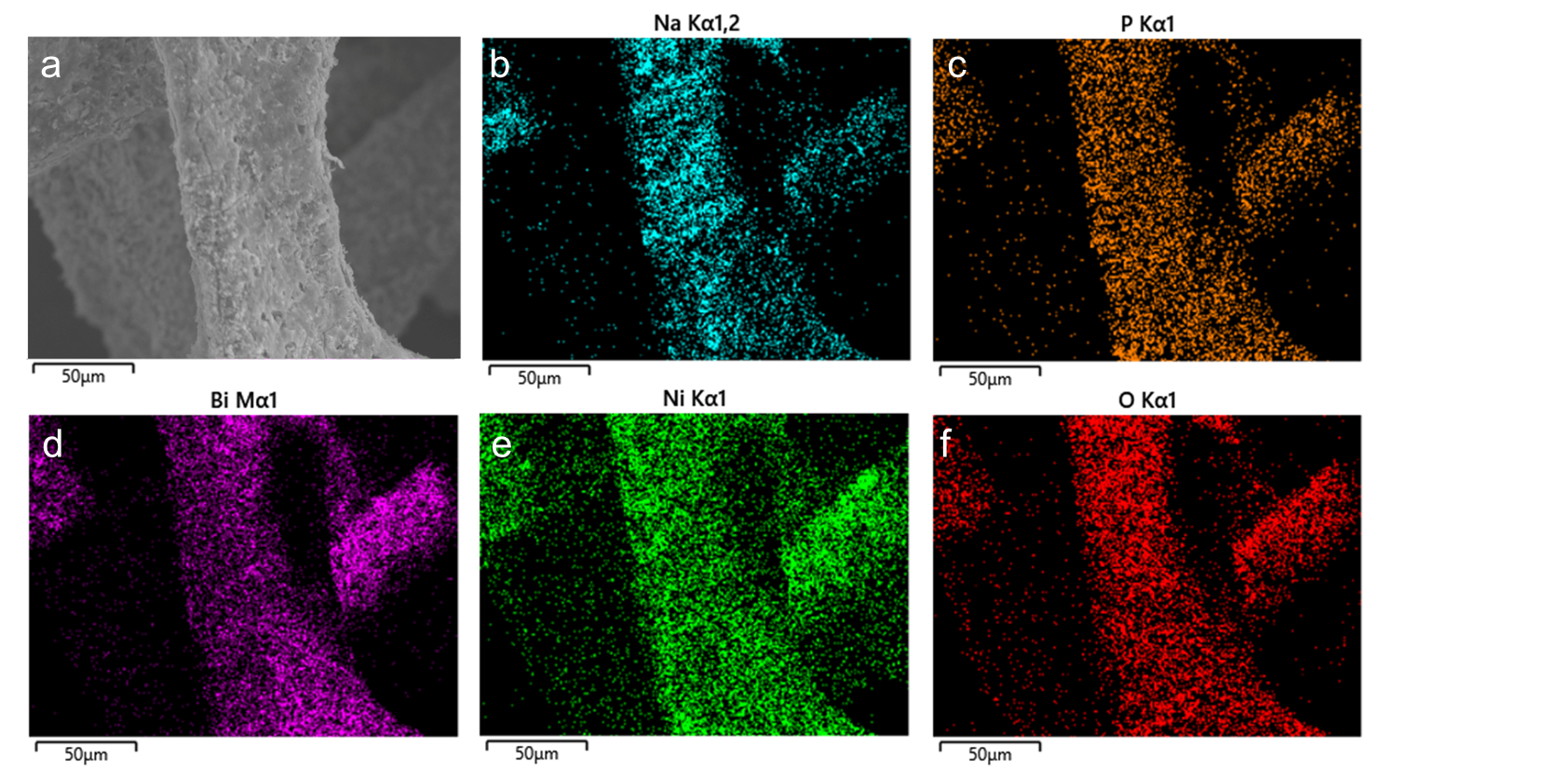


Figure S11. a) SEM image obtained for Bi-BEP electrode under open-circuit potential after 3000 cycles and corresponding energy dispersive X-ray spectroscopy (EDS) elemental mapping for b) Na; c) P; d) Bi; e) Ni; f) O.


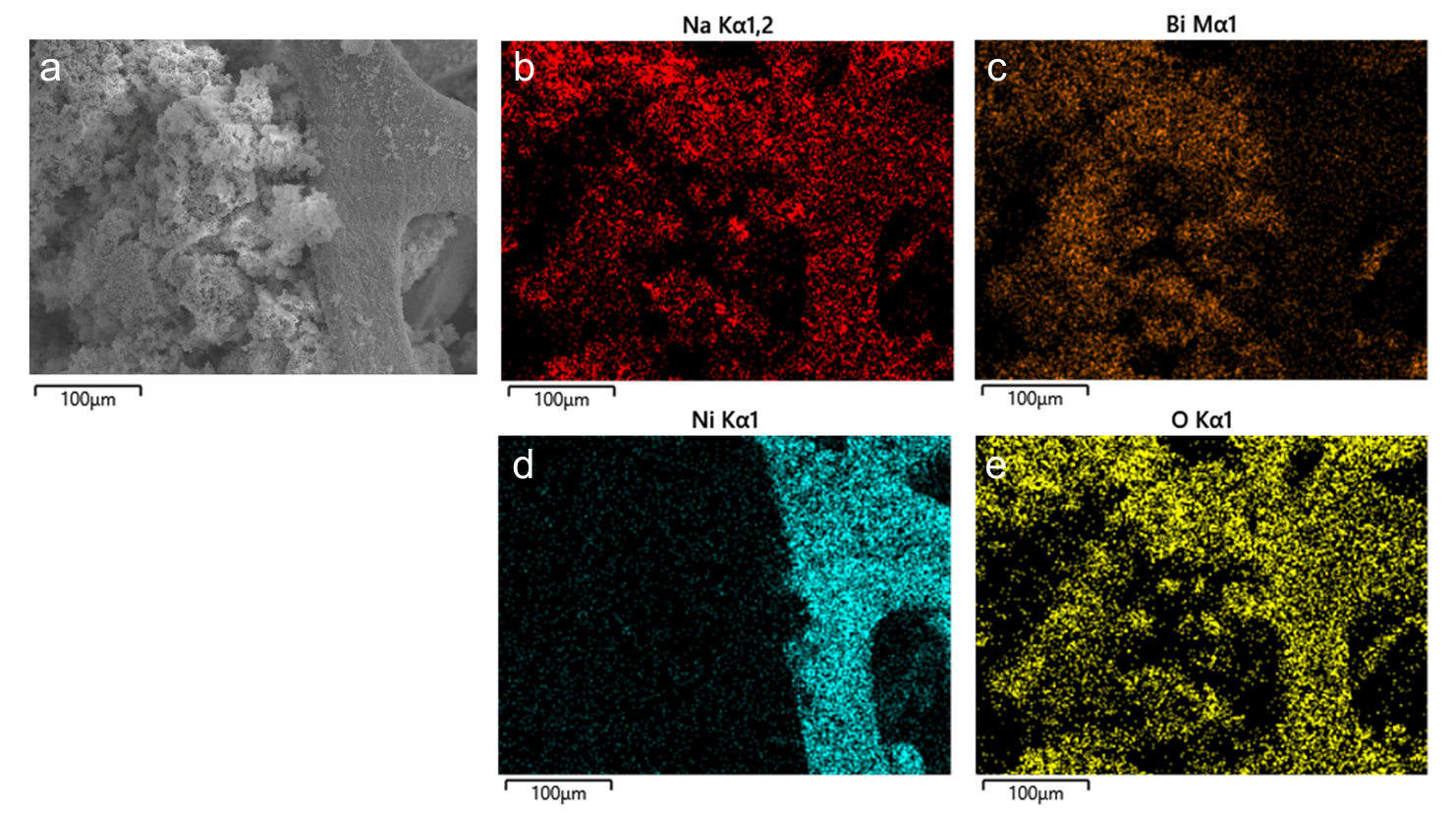


Figure S12. a) SEM image for Bi-BE electrode under open-circuit potential after 3000 cycles and corresponding EDS elemental mapping for b) Na; c) Bi; d) Ni; e) O.


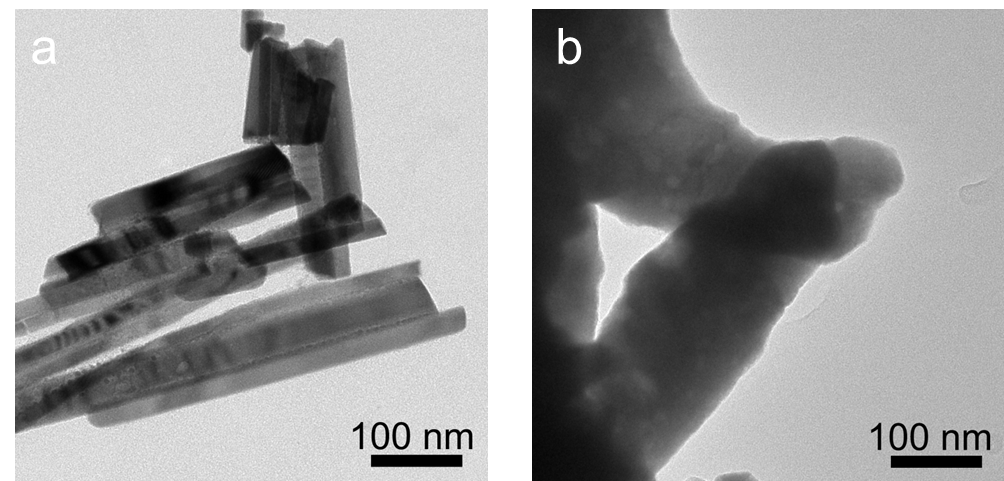


Figure S13. TEM image at charging state of −1.2 V (*vs.* SCE) for a) Bi-BE and b) Bi-BEP electrode after 200 cycles.


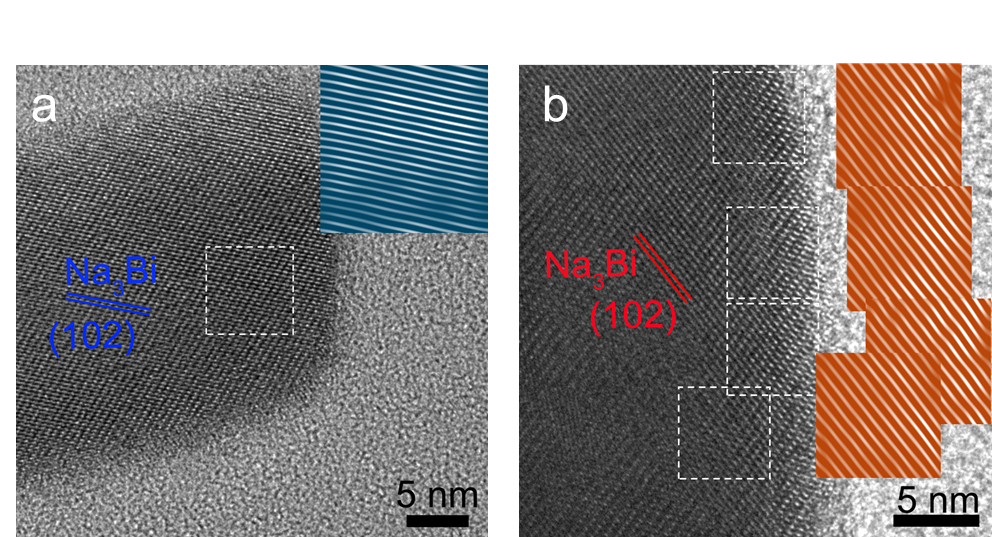


Figure S14. HRTEM image at charging state of −1.2 V (*vs.* SCE) and IFFT for a) Bi-BE and b) Bi-BEP electrode after 200 cycles.


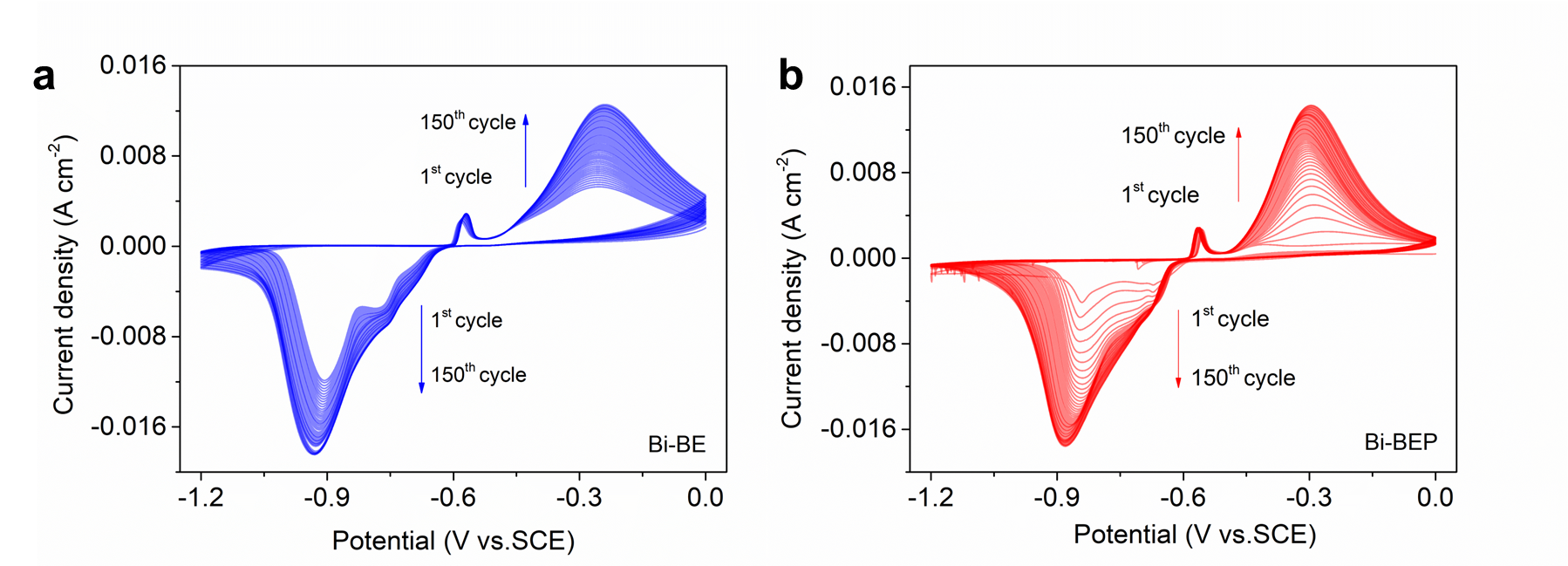


Figure S15. CV curves of a) Bi-BE and b) Bi-BEP at a scan rate of 1 mV s^−1^ for 150 cycles.


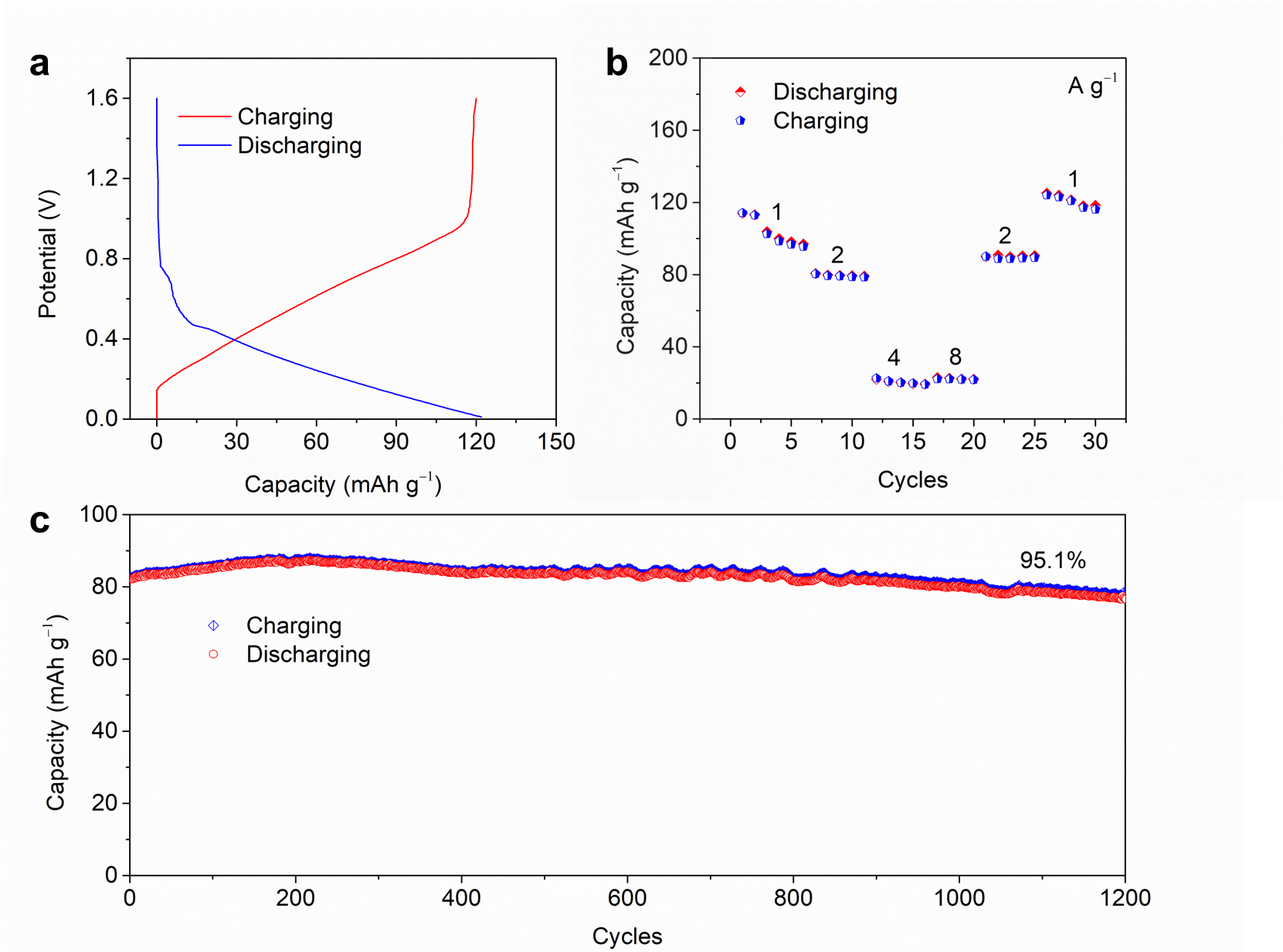


Figure S16. Electrochemical performance for Bi//AC full battery a) GCD curves at a current density of 1 A g^−1^; b) Rate performance at different current densities and c) Cycling stability at a current density 2 A g^−1^ for 1200 cycles.

To evaluate the practicality of the Bi-BEP electrode, a full Bi//AC battery with an operating voltage of 1.6 V was assembled. In this configuration, the Bi-BEP electrode served as the anode, while activated carbon (AC) acted as the cathode, with BEP electrolyte as the electrolyte. Distinct charging and discharging voltage plateaus were observed, confirming the battery-like behavior of the system (Figure. S16a). As shown in Figure. S16b, the Bi//AC battery delivered high specific capacities of 113.2, 79.6, 22.0, and 20.2 mAh g^−1^ at current densities of 1, 2, 4, and 8 A g^−1^ (based on the mass of the Bi electrode). When the current density was reverted to 1 A g^−1^, the discharge capacity recovered to 124.1 mAh g^−1^, demonstrating good rate capability and reversibility. Moreover, the Bi//AC battery exhibited exceptional cycling stability at 2 A g^−1^ (Figure. S16c), retaining 95.1% of its initial capacity (82.4 mAh g^−1^) after 1200 cycles. This outstanding electrochemical performance highlights the stabilizing effect of PO_4_^3−^ and underscores the application potential of the Bi-BEP electrode in energy storage systems.


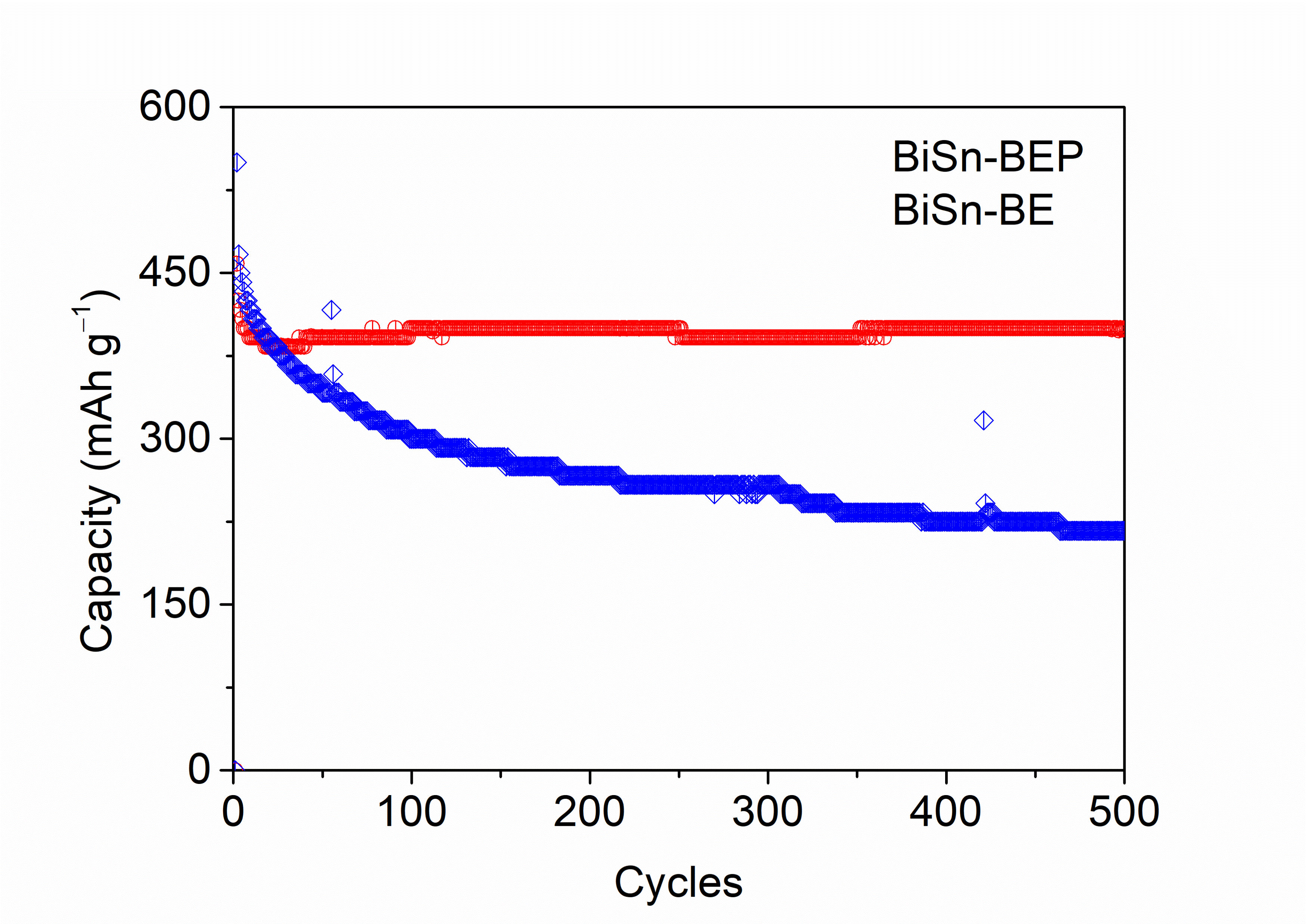


Figure S17. Cycling stability for BiSn electrode at a current density of 1 A g^−1^ in BE and BEP electrolytes.


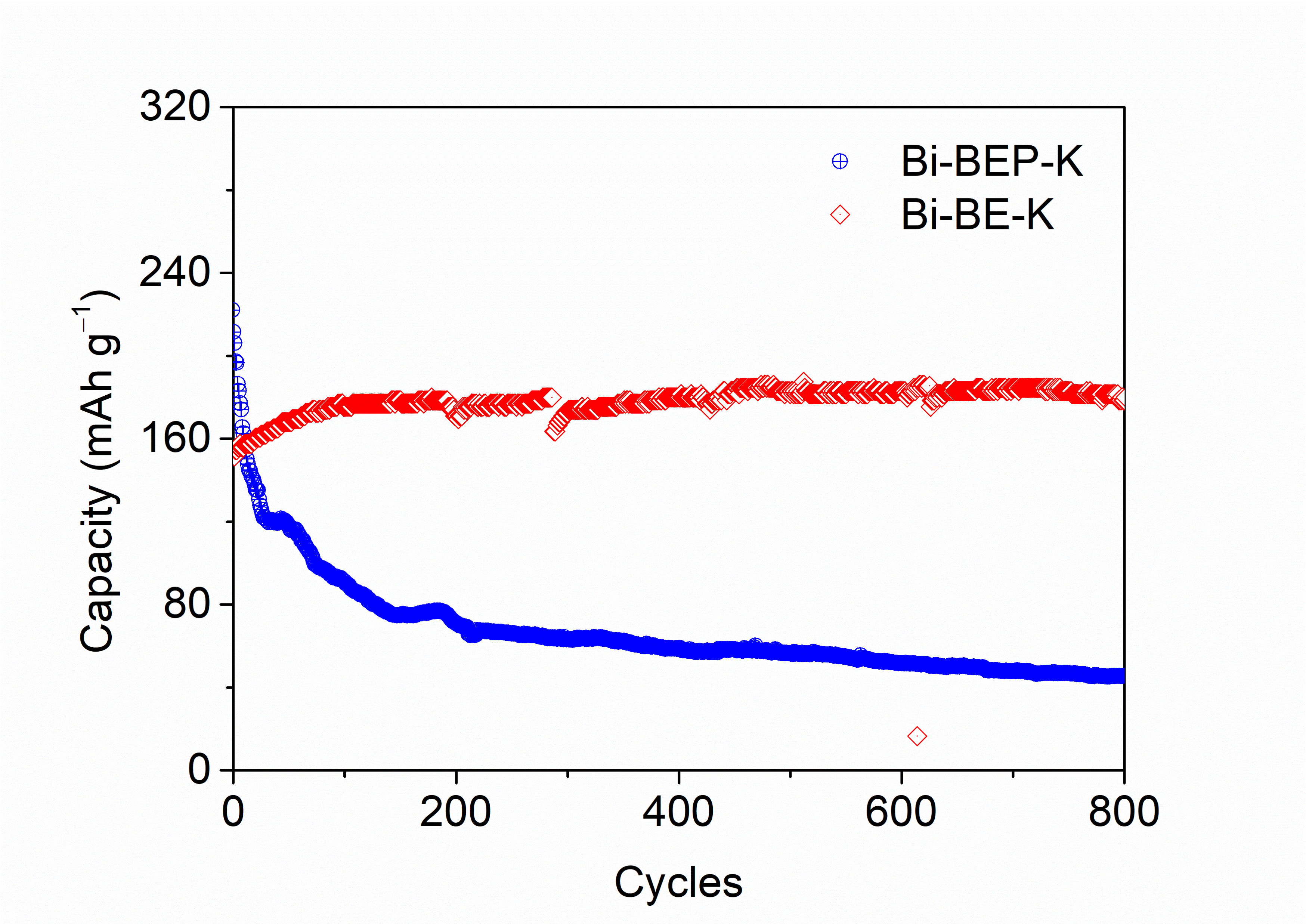


Figure S18. Cycling stability for Bi electrode at a current density of 2 A g^−1^ in BE-K and BEP-K electrolytes.


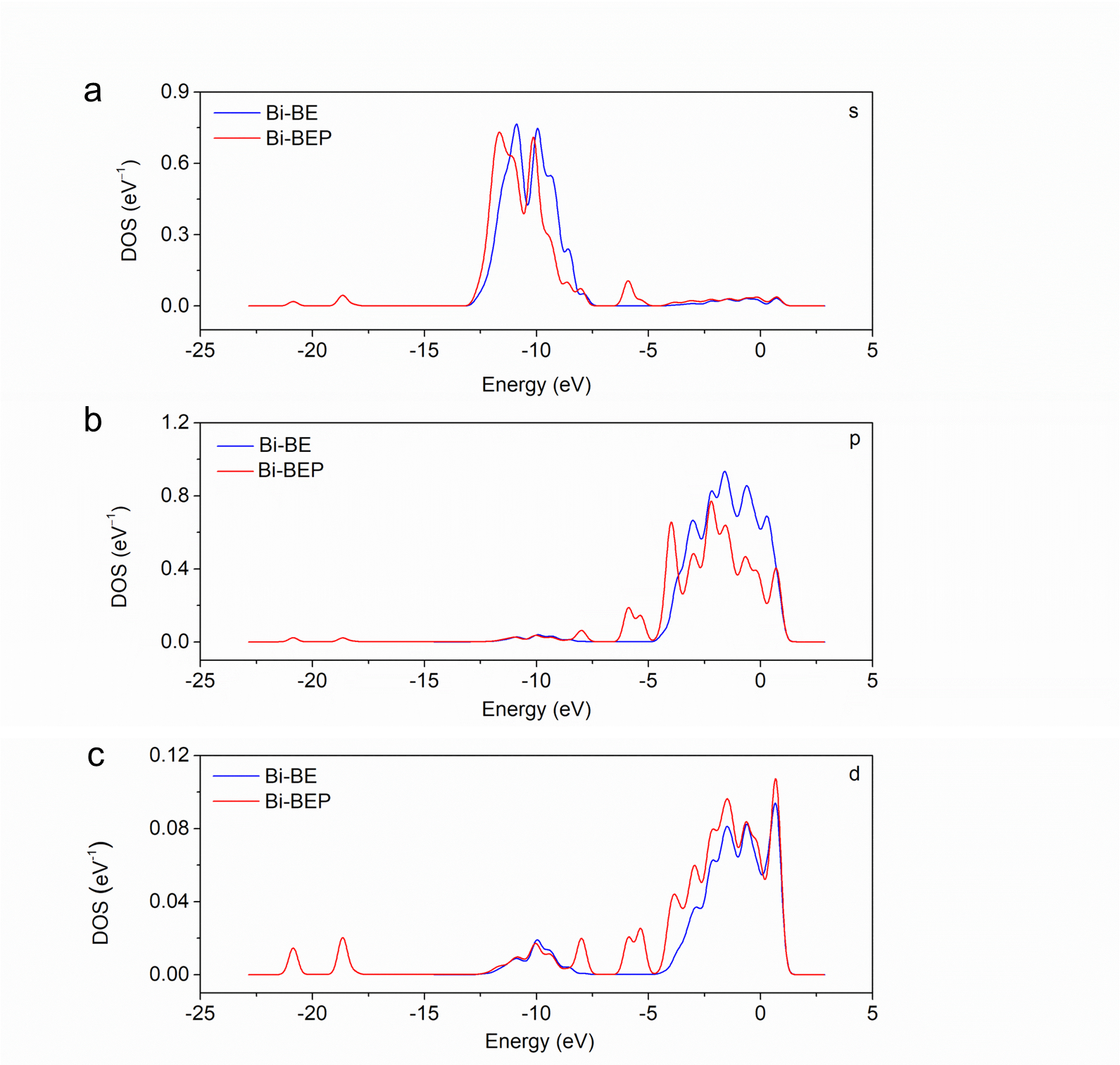


Figure S19. DOS spectra for a) s, b) p and c) d orbit for Bi-BE and Bi-BEP electrodes.


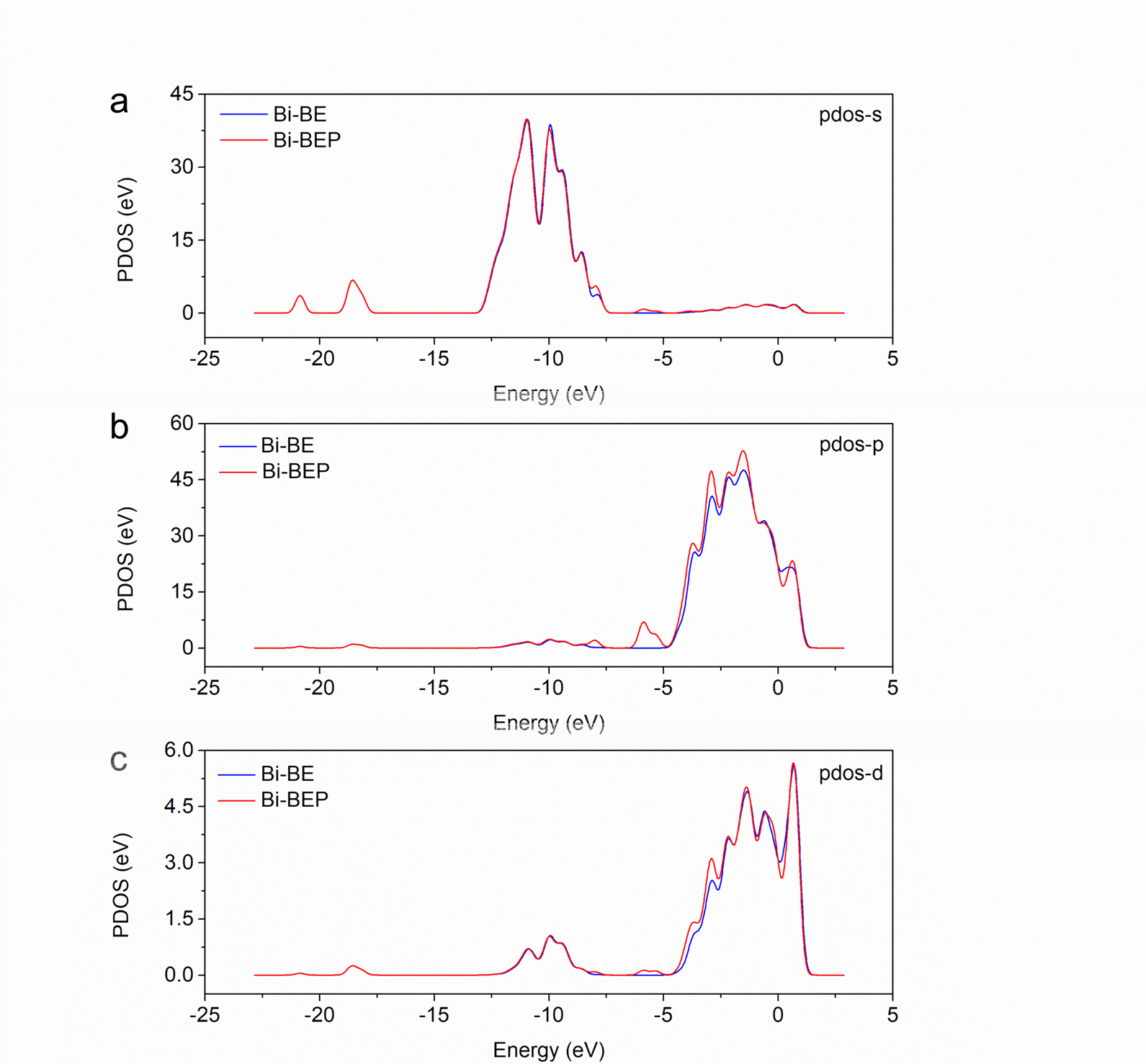


Figure S20. PDOS spectra for a) s, b) p and c) d orbit for Bi-BE and Bi-BEP electrodes.


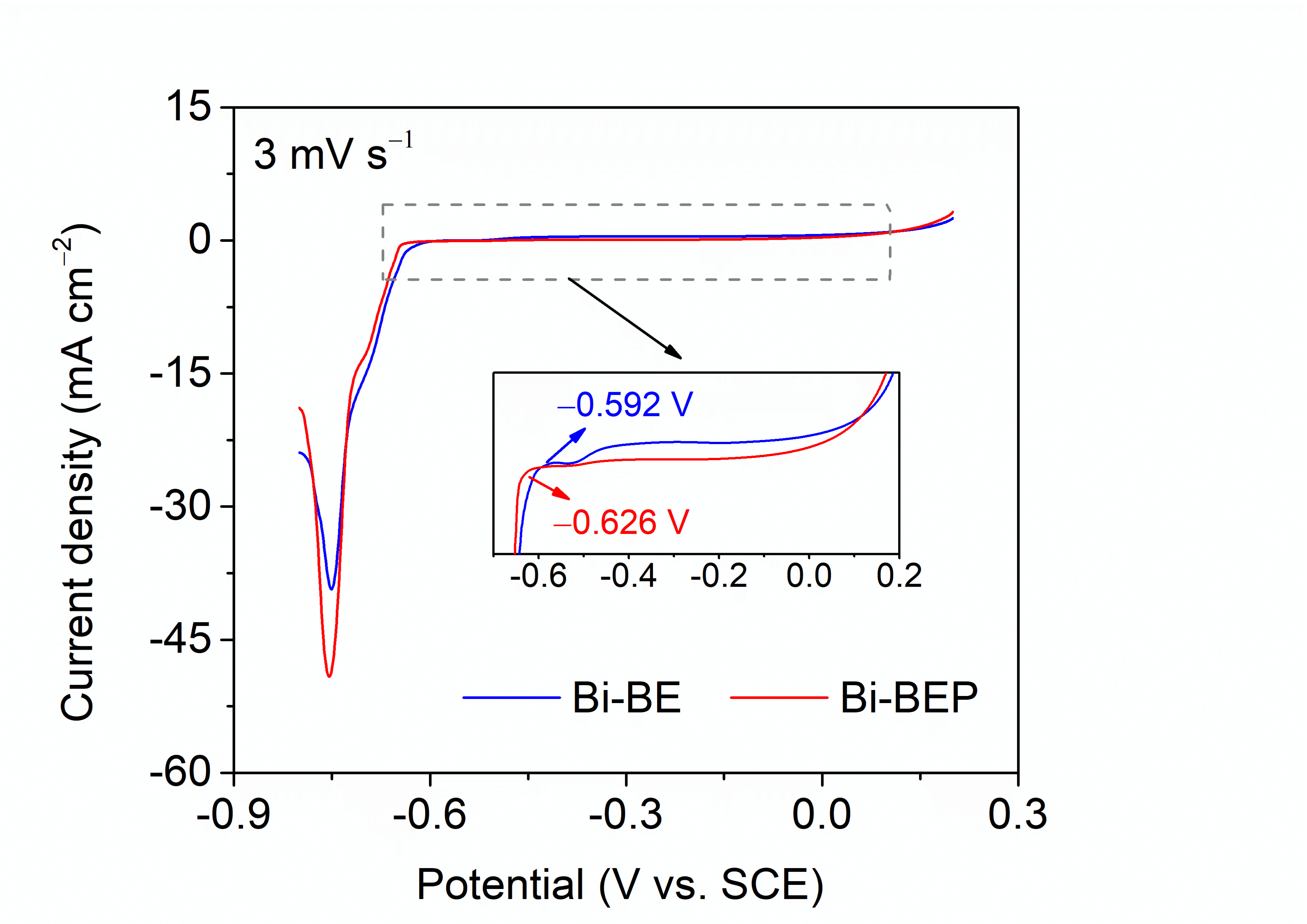


Figure S21. The linear sweep voltammetry (LSV) curves of the Bi electrode in BE and BEP electrolytes.


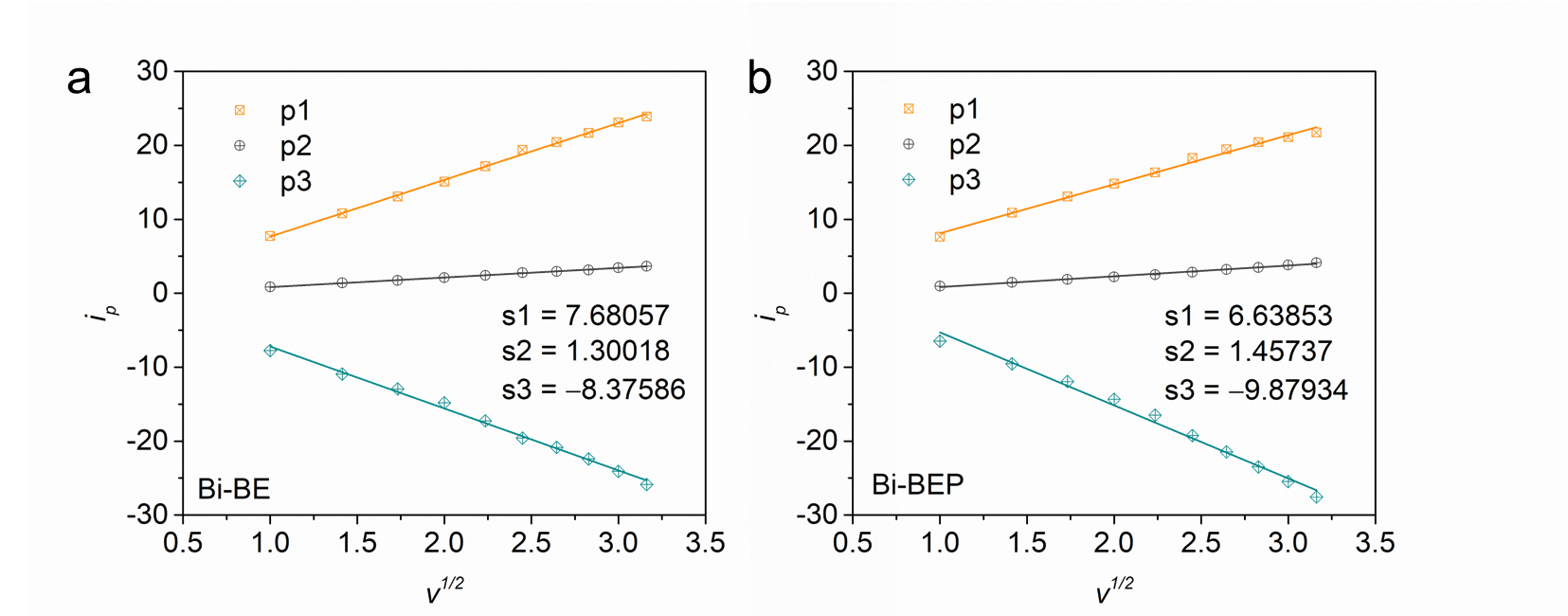


**Figure S22.** Plots of *v_1/2_ vs. i_p_* obtained from CV curves in Figure. S10 for **a)** Bi-BE and **b)** Bi-BEP electrode.

**Table S1.** Comparison of electrochemical performance of various reported Bi-Based electrode materials

| Bi-based Materials | Electrolyte | Capacity  /Current Density | Capacity Retention  /Current Density | Rate Capacity | Ref |
| --- | --- | --- | --- | --- | --- |
| BiOBr | alkaline | 53 mAh g^−1^  at 0.5 A g^−1^ | 75% after 1000 cycles  at 4 A g^−1^ | 159 mAh g^−1^  at 4 A g^−1^ | [17] |
| Bi film | alkaline | 170 mAh·g^−1^  at 0.5 A·g^−1^ | 60% after 90 cycles |  | [18] |
| rGO/Bi/CNT | alkaline | 146.9 mAh g^−1^  at 1 A g^−1^ | initially increased and then well-maintained during  10 000 cycles at 100 mV s^−1^ | 126.3 mAh g^−1^  at 25 A g^−1^ | [19] |
| Single-crystalline Bi | alkaline | 96.2 mA h [g^−1^ at 4.5](mailto:g−1@4.5) A·g^−1^ | ------- | 90.4 mAh g^−1^  at 45 A·g^−1^ | [20] |
| 2D BiOI | alkaline | 2.17 mA h cm^2^  at 1 mA cm^2^ | 93.1% after 5000 cycles at 64 mA cm^2^ (mass of 27 mg cm^2^) | 1.48 mA h cm^2^  at 128 mA cm^2^ | [21] |
| BiOBr_0⋅87_I_0.13_ | alkaline | 187.6 mAh g^−1^  at 1 A g^−1^ | 77.1 % after 100 cycles  at 1A g^−1^ | 153.1 mAh g^−1^  at 4 A g^−1^ | [22] |
| ẟ-Bi_2_O_3_ | alkaline | 264 mAhg^−1^  at 0.2 A g^−1^ | 85.6% after 1500 cycles  at 0.2 A g^−1^ | 150 mA hg^−1^  at 2 A g^−1^ | [23] |
| Bi_2_O_3_-Bi_2_S_3_ | alkaline | 0.68 mAh cm^-2^ at 4 mA cm^-2^ | 50% after 5000 cycles  at 80 mA cm^-2^ | 0.45 mAh  at 80 mA cm^−2^ | [24] |

References

[1] T. Qin, X. Chu, T. Deng, B. Wang, X. Zhang, T. Dong, Z. Li, X. Fan, X. Ge, Z. Wang, P. Wang, W. Zhang, W. Zheng, *Journal of Energy Chemistry* **2020**, 48, 21.

[2] T. Qiao, Z. Qiao, S. Sun, S. Zhou, *Journal of Computational and Applied Mathematics* **2024**, 443, 115759.

[3] a)K. Gelderman, L. Lee, S. W. Donne, *J. Chem. Educ.* **2007**, 84, 685; b)W. J. Albery, G. J. O'Shea, A. L. Smith, *J. Chem. Soc., Faraday Trans.* **1996**, 92, 4083; c)S. Park, T. Schultz, D. Shin, N. Mutz, A. Aljarb, H. S. Kang, C.-H. Lee, L.-J. Li, X. Xu, V. Tung, E. J. W. List-Kratochvil, S. Blumstengel, P. Amsalem, N. Koch, *ACS Nano* **2021**, 15, 14794.

[4] P. Simon, Y. Gogotsi, B. Dunn, *Science* **2014**, 343, 1210.

[5] G. Kresse, D. Joubert, *Phys. Rev. B* **1999**, 59, 1758.

[6] J. Perdew, K. Burke, M. Ernzerhof, *Phys. Rev. Lett.* **1996**, 77, 3865.

[7] S. Grimme, J. Antony, S. Ehrlich, H. Krieg, *The Journal of chemical physics* **2010**, 132, 154104.

[8] J. Hutter, M. Iannuzzi, F. Schiffmann, J. VandeVondele, *Wiley Interdisciplinary Reviews: Computational Molecular Science* **2014**, 4, 15.

[9] G. Lippert, J. Hutter, M. Parrinello, *Mol. Phys.* **1997**, 92, 477.

[10] J. VandeVondele, J. Hutter, *The Journal of chemical physics* **2007**, 127, 114105.

[11] S. Goedecker, M. Teter, J. Hutter, *Phys. Rev. B* **1996**, 54, 1703.

[12] F. Neese, *Wiley Interdisciplinary Reviews: Computational Molecular Science* **2012**, 2, 73.

[13] S. J. Clark, M. D. Segall, C. J. Pickard, P. J. Hasnip, M. I. Probert, K. Refson, M. C. Payne, *Z. Krist.-Cryst. Mater.* **2005**, 220, 567.

[14] T. Qin, W. Zhang, Y. Ma, W. Zhang, T. Dong, X. Chu, T. Li, Z. Wang, N. Yue, H. Liu, L. Zheng, X. Fan, X. Lang, Q. Jiang, W. Zheng, *Energy Storage Materials* **2022**, 45, 33.

[15] Q. Hao, R. Wang, H. Lu, C. a. Xie, W. Ao, D. Chen, C. Ma, W. Yao, Y. Zhu, *Applied Catalysis B: Environmental* **2017**, 219, 63.

[16] C. Pan, J. Xu, Y. Wang, D. Li, Y. Zhu, *Advanced Functional Materials* **2012**, 22, 1518.

[17] H. Liu, T. Y. K. Qin, Y. Zhang, X. Zhao, W. Dong, Z. Chang, S. K. W. Leong, Z. Rafa, M. Ni, D. Y. C. Leung, W. Pan, *Journal of Energy Chemistry* **2025**, 103, 749.

[18] W. Zuo, P. Xu, Y. Li, J. Liu, *Nanomaterials*, 10.3390/nano5041756

[19] M. Wang, S. Xie, C. Tang, Y. Zhao, M. Liao, L. Ye, B. Wang, H. Peng, *Advanced Functional Materials* **2020**, 30, 1905971.

[20] Y. Zeng, Z. Lin, Y. Meng, Y. Wang, M. Yu, X. Lu, Y. Tong, *Advanced Materials* **2016**, 28, 9188.

[21] P. Liu, X. Zhang, L. Feng, H. Pan, B. Yang, X. Zheng, G. Dong, *Journal of Materials Chemistry A* **2021**, 9, 15472.

[22] T. Qin, L. Zhou, J. Fu, T. Dong, Y. Han, Y. Qiao, X. Wang, Z. Guo, *Journal of Power Sources* **2024**, 613, 234814.

[23] T. Qin, X. Zhang, D. Wang, T. Deng, H. Wang, X. Liu, X. Shi, Z. Li, H. Chen, X. Meng, W. Zhang, W. Zheng, *ACS Applied Materials & Interfaces* **2019**, 11, 2103.

[24] Q. Li, J. Fu, L. Zhang, W. Zhang, X. Wang, Y. Feng, H. Fu, Z. Yong, J. Guo, K. Tian, C. Liu, W. Gong, *ACS Applied Materials & Interfaces* **2024**, 16, 36413.
